# GLT-1-Dependent Disruption of CNS Glutamate Homeostasis and Neuronal Function by the Protozoan Parasite *Toxoplasma gondii*

**DOI:** 10.1371/journal.ppat.1005643

**Published:** 2016-06-09

**Authors:** Clément N. David, Elma S. Frias, Jenny I. Szu, Philip A. Vieira, Jacqueline A. Hubbard, Jonathan Lovelace, Marena Michael, Danielle Worth, Kathryn E. McGovern, Iryna M. Ethell, B. Glenn Stanley, Edward Korzus, Todd A. Fiacco, Devin K. Binder, Emma H. Wilson

**Affiliations:** 1 Division of Biomedical Sciences, School of Medicine, University of California, Riverside, Riverside, California, United States of America; 2 Department of Psychology, University of California, Riverside, Riverside, California, United States of America; 3 Department of Neuroscience, University of California, Riverside, Riverside, California, United States of America; Johns Hopkins University School of Medicine, UNITED STATES

## Abstract

The immune privileged nature of the CNS can make it vulnerable to chronic and latent infections. Little is known about the effects of lifelong brain infections, and thus inflammation, on the neurological health of the host. *Toxoplasma gondii* is a parasite that can infect any mammalian nucleated cell with average worldwide seroprevalence rates of 30%. Infection by *Toxoplasma* is characterized by the lifelong presence of parasitic cysts within neurons in the brain, requiring a competent immune system to prevent parasite reactivation and encephalitis. In the immunocompetent individual, *Toxoplasma* infection is largely asymptomatic, however many recent studies suggest a strong correlation with certain neurodegenerative and psychiatric disorders. Here, we demonstrate a significant reduction in the primary astrocytic glutamate transporter, GLT-1, following infection with *Toxoplasma*. Using microdialysis of the murine frontal cortex over the course of infection, a significant increase in extracellular concentrations of glutamate is observed. Consistent with glutamate dysregulation, analysis of neurons reveal changes in morphology including a reduction in dendritic spines, VGlut1 and NeuN immunoreactivity. Furthermore, behavioral testing and EEG recordings point to significant changes in neuronal output. Finally, these changes in neuronal connectivity are dependent on infection-induced downregulation of GLT-1 as treatment with the ß-lactam antibiotic ceftriaxone, rescues extracellular glutamate concentrations, neuronal pathology and function. Altogether, these data demonstrate that following an infection with *T*. *gondii*, the delicate regulation of glutamate by astrocytes is disrupted and accounts for a range of deficits observed in chronic infection.

## Author Summary

The protozoan parasite *Toxoplasma gondii* infects a third of the world’s population and causes a chronic lifelong infection in the brain of the host. The consequences of such an infection are poorly understood. Here, we demonstrate that *Toxoplasma* infection can induce profound changes in astrocyte physiology leading to significant disruption of neuronal networks. Pathology can be rescued by upregulating the astrocytic glutamate transporter, GLT-1, restoring concentrations of extracellular glutamate and EEG power. We suggest that such global dysregulation of neurotransmitters should be considered when determining the effects of infection on the CNS.

## Introduction

The balancing act required to fight infection while maintaining tissue homeostasis is perhaps no more critical than in the CNS. Here, the physical restraints imposed by the skull and blood brain barrier alongside low MHC expression and a lack of circulating lymphocytes can delay and limit the immune response. However, perhaps due to these properties, many infectious agents target or localize to the brain [1]. These include bacterial, viral and parasitic pathogens. In many cases, such infections are chronic and require immune competency to remain latent [2–4]. We have only nominal understanding of the effects of continuous immune reactivity in a tissue that is designed to keep such responses to a minimum.

Recently, the concept that infections can have a profound and lasting effect on brain function and even host behavior has gained momentum [1, 3, 4]. In the case of the protozoan parasite *Toxoplasma gondii*, this lifelong infection resides predominantly as latent cysts inside neurons and requires continuous infiltration of lymphocytes to the brain to prevent parasite reactivation and fatal encephalitis [2, 5]. Furthermore, this is not a selective parasite: *Toxoplasma* is one of the most successful pathogens on the planet infecting approximately a third of the world’s population with prevalence rates in Europe and the USA between 15–80% [6]. Although infection is common, disease induced by *Toxoplasma* is rare and with few exceptions has only been associated with individuals that have profound defects in their immune system. However, in recent years, interest has focused on the potential relationship of infection with *Toxoplasma* on neurodegenerative and psychiatric disorders. Infection with *Toxoplasma* has been suggested to be a significant risk factor in Parkinson’s [7], Alzheimer’s (AD) [8], mania [9] and Schizophrenia [10–12] and is correlated with specific changes in murine [13–15] and human behavior [16, 17]. The range of severity and the variety of disease associated with *Toxoplasma* infection suggest the potential for global, as well as specific, alterations in neuronal networks and signaling.

Glutamate is arguably the most important neurotransmitter in the brain and unregulated levels can cause neuroexcitotoxicity; therefore, CNS glutamate is strictly controlled. Although glutamate is readily available in the periphery, it does not cross the BBB and is thus virtually completely synthesized *de-novo* within the CNS, primarily by astrocytes [18–20], with neurons able to catalyze glutamine to glutamate through glutaminase. Astrocytes have the important job of regulating CNS glutamate by adjusting uptake, release, synthesis into glutamine, and synthesis from α-ketoglutarate or lactate/alanine [18–21]. These cells play an active role in controlling *Toxoplasma* infection with the ability to up-regulate pro-inflammatory cytokines, secrete chemoattractants and internally kill parasites via IGTP [22–24]. However, observations of astrocytic swelling during infection [25] may point to significant changes in astrocyte physiology. Such changes in astrocyte activation and morphology have been linked to dysregulation in glutamate metabolism either via slowing glutamate clearance or dumping of glutamate into the extracellular space (ECS)[26–32]. The implications of glutamate excitotoxicity are broad. Traditionally, elevated extracellular glutamate concentrations have been observed under acute CNS insults such as ischemia [33, 34] and traumatic brain injury [35, 36]. However, many studies have associated elevated ECS glutamate concentrations with neurodegenerative disorders such as amyotrophic lateral sclerosis (ALS) [37], multiple sclerosis (MS) [38], Alzheimer’s disease [39, 40] and with CNS infections such as cerebral malaria (CM) [41]. Understanding the mechanisms that underlie elevated ECS concentrations of glutamate following CNS insults is critical in developing preventative measures to avoid neuronal death. If there are changes in glutamate concentrations, then this could lead to major changes in neuronal biology in the brain.

In these studies, we use microdialysis in the preferentially infected frontal cortex to directly collect and measure extracellular concentrations of glutamate. Although cells may contain high levels of intracellular glutamate, it is the increase in extracellular glutamate that can cause neuronal damage. We demonstrate that during infection with *Toxoplasma*, astrocyte glutamate regulation is disrupted and CNS extracellular levels of glutamate reach non-homeostatic ranges. Consistent with a dysregulation of glutamate, we reveal a pattern of neurological damage including decreases in ß-III tubulin, VGlut1 expression, density of dendritic spines and functional output as measured by EEG analysis. Thus, this infection, and the resulting inflammation, leads to dysregulation of glutamate control by astrocytes resulting in neuronal pathology and previously undocumented loss of EEG power. Finally, damage to neuronal health is rescued by upregulation of the glutamate transporter GLT-1 following the administration of ceftriaxone. The significance of these results suggest that in contrast to assuming chronic *Toxoplasma* infection as quiescent and benign, we should be aware of the significant risk to normal neurological pathways and changes in brain chemistry.

## Results

### Infection induces chronic astrocytic morphological and molecular changes

**Fig 1:**
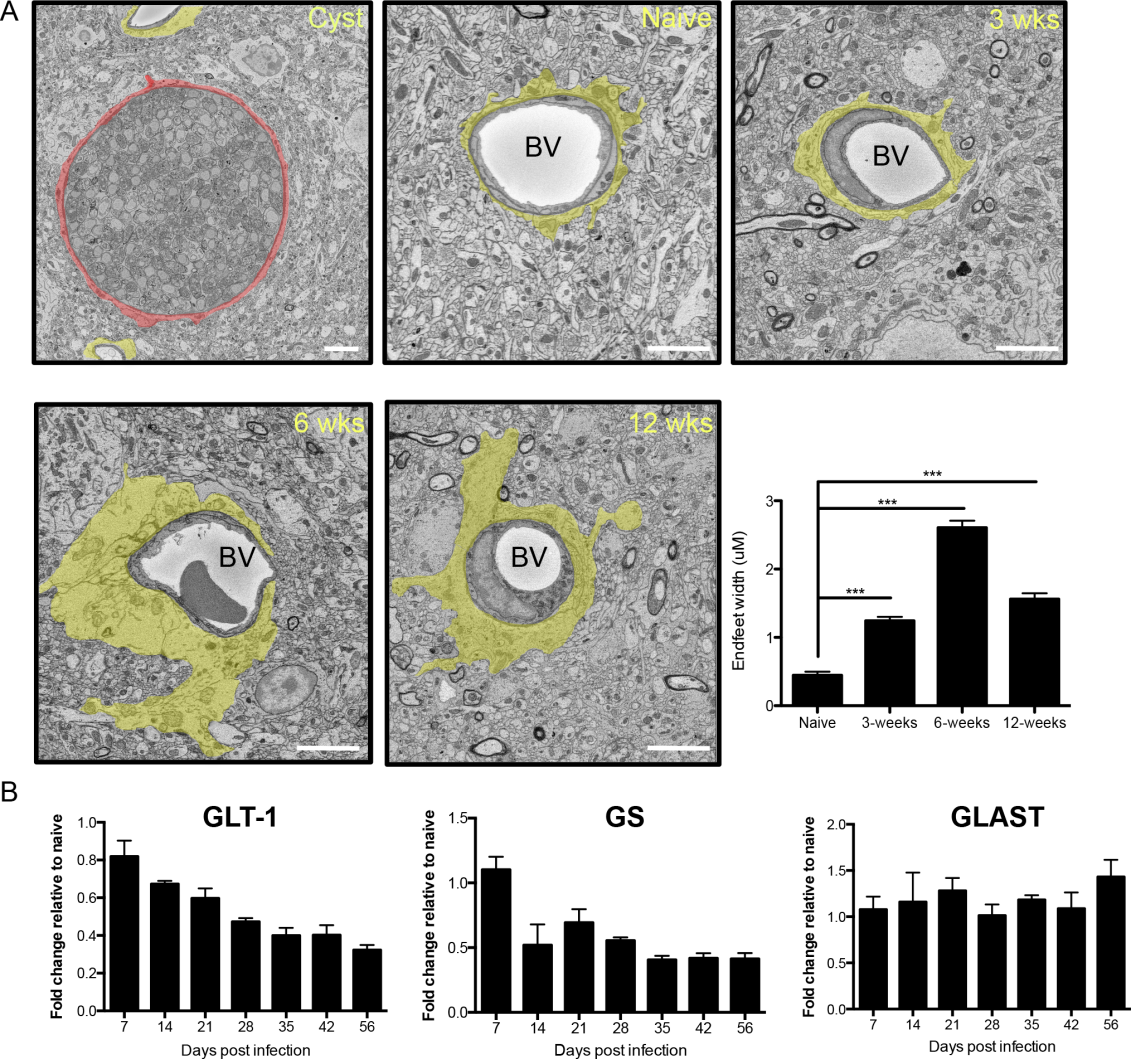
Infection induces chronic astrocytic morphological and molecular changes. C57Bl/6 mice were infected with *Toxoplasma* and brains harvested. A) Scanning serial electron microscopy images analyzed for astrocytic endfeet width (highlighted in yellow). Quantification of endfoot width performed over the course of infection (scale bar: 5μm, BV: blood vessel). 6–10 Z stacks containing blood vessels (naïve n = 20; 3 weeks n = 75; 6 weeks n = 134; 12 weeks n = 82) 5–6μm wide were selected and average astrocyte endfoot width was quantified by measuring perivascular astrocyte area and dividing by the blood vessel circumference. Significance compared to naïve *** = p<0.001. The first panel depicts a *Toxoplasma* cyst inside a neuron (red) within the frontal cortex (unshaded micrographs provided in S1) B) RT-qPCR was performed on whole forebrain RNA with primers for GLT-1, glutamine synthetase (GS) and GLAST over the course of infection and is presented as fold increase over naïve.

Astrocyte activation has been previously observed following *Toxoplasma* infection, and indeed occurs prior to the entry of parasites to the brain [25, 42, 43]. To further assess astrocytic health during chronic infection, a series of morphological and molecular analyses were performed. To quantify the degree of morphological change and examine the kinetics of this response, astrocytic endfeet from the prefrontal cortex were analyzed from serial transmission electron microscopy images from mice infected with *Toxoplasma* over the course of chronic infection. The beginning of the chronic stage of infection is characterized by an increase in cyst burden, CNS inflammation, and a decrease in the free tachyzoite form of the parasite and occurs around the third week post infection. Sites of infection are unevenly distributed in the brain, therefore we sampled at least 10 different areas throughout the frontal cortex. On at least one occasion a cyst was observed where the surrounding tissue is clearly distorted **(Figs 1A and S1)**, otherwise endfoot measurements were made independent of parasite location. For frequent and consistent measurements, astrocytic processes enveloping only blood capillaries (~5 μm in diameter) were sampled. In the naïve brain, astrocytic endfeet are on average less than 0.5μm wide (**Figs 1A and S1**)[44]. By 3 weeks post-infection, astrocytic endfeet are already significantly swollen. At 6 weeks post infection, well into the chronic phase, cyst burden has reached a plateau [45], and the immune response and parasite reactivation have reached a stable balance [25]. Here, astrocytic endfeet width peaks to almost 6 fold higher than naïve (2.606± 0.1021μm; p<0.0001) **(Figs 1A and S1)**. The balance between cyst burden and the immune response is maintained for the remainder of the host’s life. To test if astrocytic endfeet remain swollen, endfeet were measured at 12 weeks post infection, late in the chronic phase [25]. Although the endfeet width decreases to 1.562± 0.0840μm, they remain significantly swollen compared to naïve animals (p<0.0001) **(Figs 1A and S1)**. This supports the previously observed swelling of astrocytes following infection, but in addition demonstrates that this is a chronic and sustained state pointing instead to a hypertrophic condition rather than transient swelling.

This type of change in morphology is frequently indicative of significant changes in the molecular and functional role of these cells. One of the major roles of astrocytes is to remove extracellular glutamate to prevent neuroexcitotoxicity [21]. In the brain, all glutamate is synthesized *de-novo* as no transporters exist on the BBB to facilitate entry of glutamate from the blood [18]. Glutamate is then released at presynaptic sites during synaptic transmission. This extracellular glutamate is regulated by the actions of specific transporters present on astrocytes. The main astrocytic glutamate transporter in the forebrain is GLT-1, with GLAST playing a less significant role [20]. Once glutamate is taken up by the astrocyte, glutamine synthetase (GS) converts a portion to glutamine, which can then be safely released back to neurons for conversion to glutamate. Although peripheral inflammatory events including the circulation of high concentrations of the cytokines IFN-γ and TNF-α are already occurring one-week post infection, GS and GLAST transcripts are not significantly different than naïve levels and GLAST transcription remains unaltered **(Fig 1B)**. At 2 weeks post infection, coincident with parasites entering the CNS, a decrease in GS transcripts is observed. This reduction is maintained throughout the chronic and late chronic stages of infection. In contrast, GLT-1 transcripts are significantly reduced at day 7 and continue to decline over the course of infection **(Fig 1B)**. These data point to a major defect in the ability of astrocytes to regulate extracellular glutamate concentrations throughout chronic infection.

### Toxoplasma infection causes significant neuronal pathology

**Fig 2:**
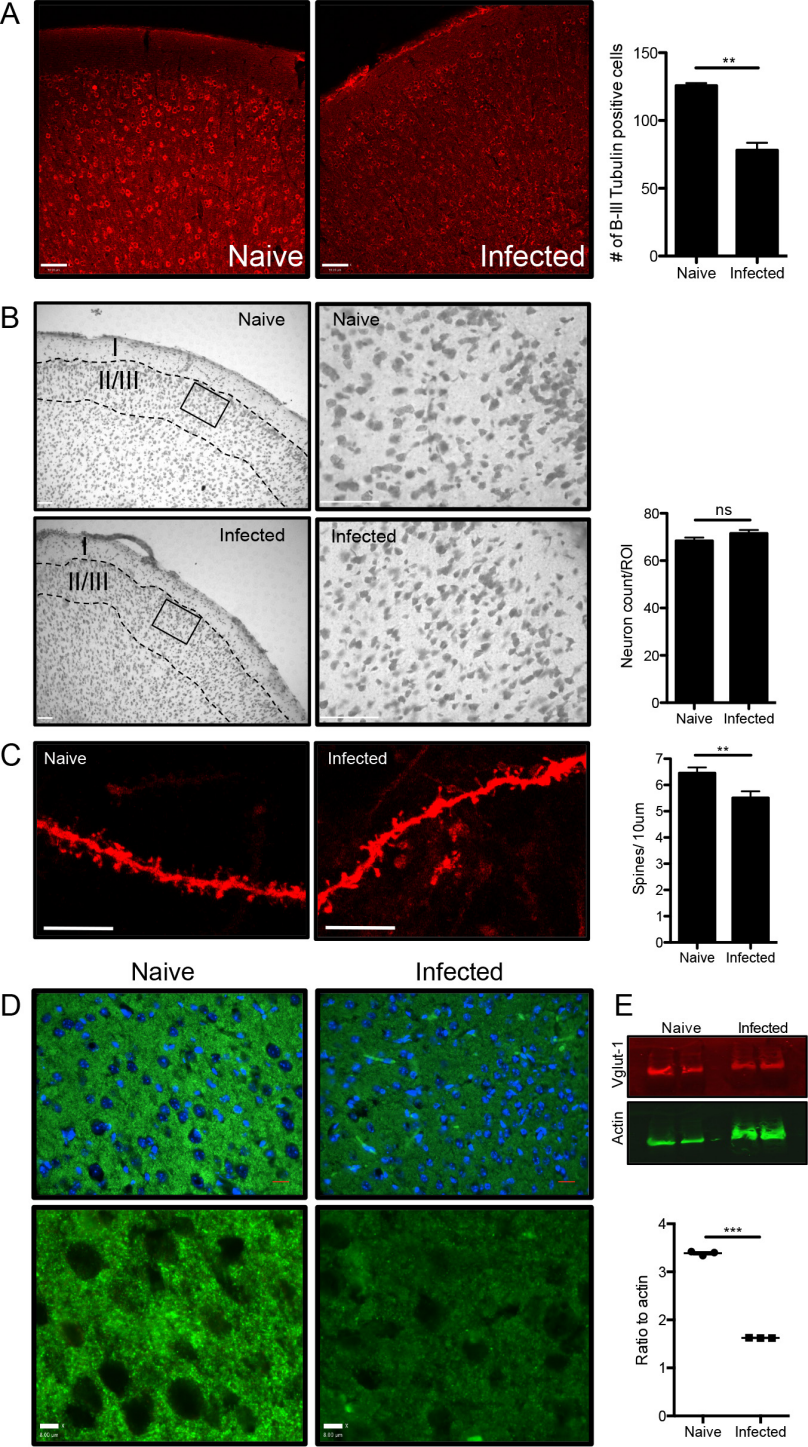
Infection with *Toxoplasma* causes neuronal pathology. Brain sections from naïve (n = 3) and 6 week infected (n = 3) mice were immunohistochemically stained for neuronal morphological markers A) 12-micron sections stained for ß-III Tubulin and quantified (p = 0.0012, scale bar: 90μm). B) Nissl staining of 40μm sections. Cortical layers labeled with roman numerals and representative ROI shown (square) and quantified (naïve: 34 ROIs, 1982 neurons; infected: 35 ROIs, 2073 neurons; p = 0.132) (scale bar 70μm and 18μm for ROI). C) Dendritic spines in the pre-frontal cortex were stained using the lipophilic dye DiI, and quantified (p = 0.0046) (scale bar: 7μm). D) VGlut-1 on 12μm sections of the frontal cortex (Scale bar: 15μm; insert scale bar: 8μm). E) Western blot and quantification of VGlut-1 from whole brain lysates (n = 3). All quantification was conducted blindly using Volocity software and significance tested using Student’s t-test ns = not significant; *** = p<0.001; ** = p<0.01.

The prefrontal cortex plays a large role in innate fear, anxiety and decision-making and is also the area of the brain most densely infected and a significant site of inflammation [46–50]. To understand the root of behavioral changes induced by infection, the prefrontal cortex was examined at 6 weeks post infection, for changes in neuronal morphology associated with excitotoxicity [47]. Immunohistochemistry staining for ß-III tubulin, a cytoskeleton component of neurons, reveals a disruption in neuronal structure. Mice infected with *Toxoplasma* exhibit significantly fewer ß-III tubulin positive cells (p = 0.0012) **(Fig 2A)**. To test if this lack of ß-III tubulin was due to the absence of neurons and therefore neuronal death or merely an indicator of poor neuronal health, Nissl staining was performed. Neuronal counts in layer II/III of the prefrontal cortex of naïve (n = 1982 neurons) and infected (n = 2073 neurons) brains reveal no significant differences in neuron density, suggesting little induction of cell death (p = 0.132)**(Fig 2B)**. Structures susceptible to excitatory damage but critical for neuronal function include dendritic spines: the postsynaptic sites of excitatory synapses. To test if synaptic changes in the frontal cortex take place during infection with *Toxoplasma*, labeling of dendritic spines was performed using the lipophilic dye, DiI delivered on tungsten particles by gene gun. Secondary dendrites in layers II/III and IV were randomly selected from serial sections of the frontal cortex and spines counted blindly. Infection with *Toxoplasma* significantly reduces spine density in 6 week infected C57BL/6 mice compared to naïve, uninfected age-matched controls (infected = 5.510±0.2474 spines per 10 μm and naive = 6.456±0.2122 spines per 10 μm; p = 0.0046) **(Fig 2C)**. Such changes are on par with neurodisorders such as Autism spectrum and Fragile X, disease conditions that are associated with a loss of synapses in the brain contributing to impaired cognitive abilities [51–53]. To further test the extent of synaptic loss, IHC staining for the excitatory presynaptic marker VGlut-1 was conducted and reveals a loss of VGlut-1 positive presynaptic boutons in the cortex of chronically infected animals. Western blot analysis of whole brain extracts supports such a downregulation, demonstrating a significant decrease in the amount of VGlut-1 in infected animals **(Fig 2D and 2E)**. These data demonstrate that although the infection is categorized as asymptomatic, with no neuronal death observed, significant disruption in neuronal cytoskeleton and significant decreases in excitatory synapses occur during the chronic phase of *Toxoplasma* infection.

### Glutamate extracellular concentrations increase during infection

**Fig 3:**
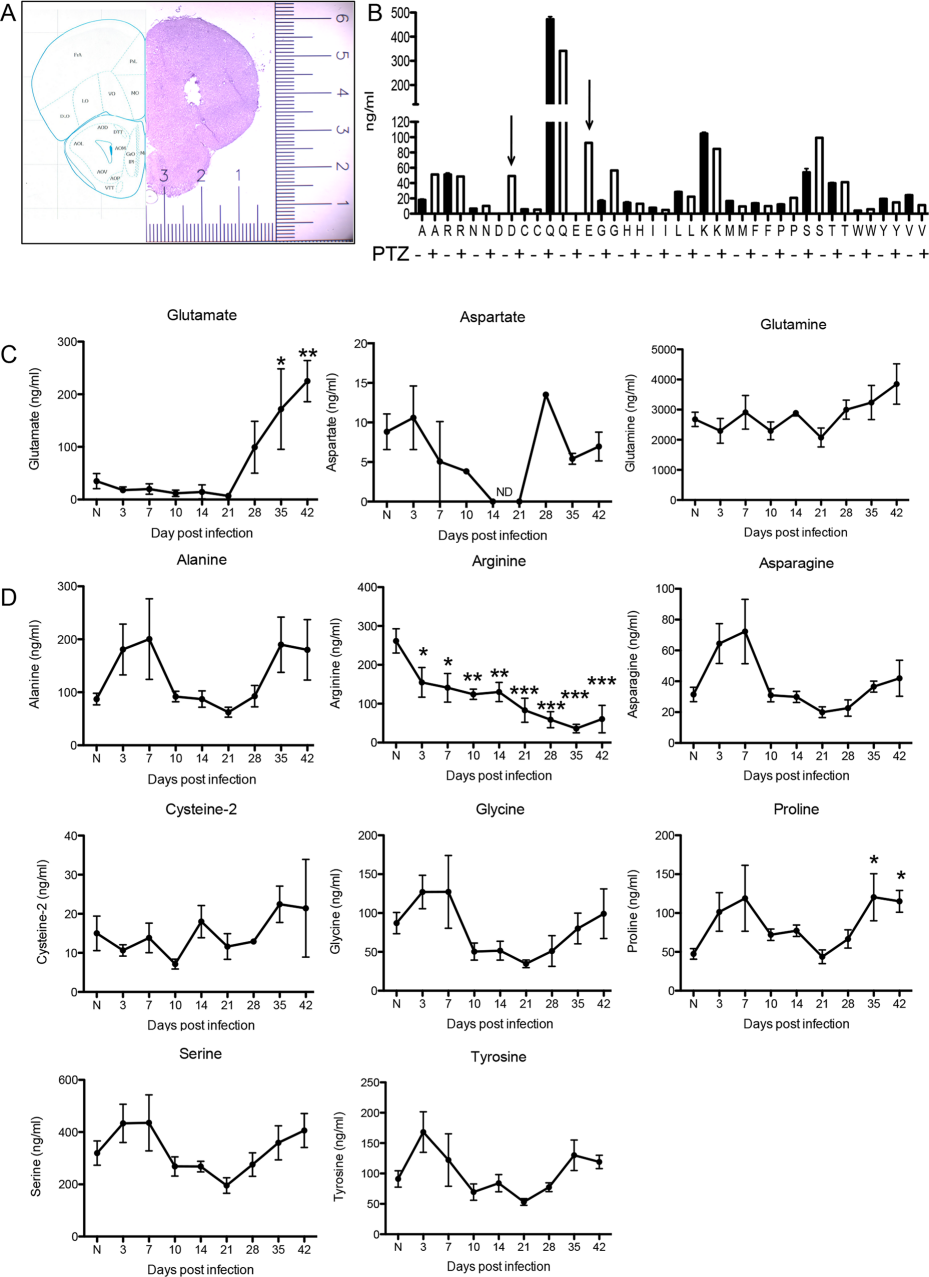
Glutamate extracellular concentrations increase during infection. Microdialysis was performed over the course of *Toxoplasma* infection taking measurements prior to (N) and after infection as indicated (n = 13 biological replicates (3 prior to infection; 2 for each time point thereafter)). A) Hematoxylin and eosin staining of microdialysis probe placement in the frontal cortex. B) Intraperitoneal injections of pentylenetetrazol (PTZ) to determine sensitivity of amino acid (*A-V; arrows*) detection. C, D) LC-MS analysis on microdialysis samples over the course of infection. One-way ANOVA: Glutamate (p = 0.0003), Arginine (p<0.0001), Proline (p = 0.0168), Serine (p = 0.04), and Tyrosine (p = 0.0149). A Dunnett’s post-test was performed for all timepoints against naïve concentrations and significance shown as asterisks. ‘ND’ indicates samples were below the limits of detection. Amino acids not listed did not change significantly. Essential amino acids are displayed in S2 Fig.

Global disruption of the cytoskeletal component β-III-tubulin and loss of dendritic spines may be a result of glutamate excitotoxicity. Excitotoxicity occurs when extracellular levels of glutamate increase to a pathological level, ~30μM is generally thought to be a threshold for pathology with >50μM causing significant cell death. Although direct in vivo measurement of ECS glutamate is rarely conducted [33, 39, 41], it is implicated in animal models of highly pathological and neurodegenerating diseases including multiple sclerosis, amyotrophic lateral sclerosis (ALS) [37–41] and epilepsy [54]. To measure extracellular levels of glutamate during infection with *Toxoplasma*, 1mm long microdialysis probes were positioned in the frontal cortex of C57BL/6 mice and the dialysate was collected from the same mouse cohort over the course of infection including acutely at day 3, 7 and 10 days post infection and then weekly at day 14 for a further 4 weeks with naïve, uninfected age matched controls sampled at each time point (N). At the conclusion of the experiment, brains were harvested and histologically assessed for appropriate probe positioning and signs of necrotic tissue, abscesses or hemorrhages **(Fig 3A)**. No necrosis or damage to tissue was observed other than that directly caused by probe placement. Using LC-MS, the extracellular concentration of all 20 essential and non-essential amino acids was measured. As a positive control, a separate cohort was injected intraperitoneally with pentylenetetrazol (PTZ) (60mg/kg) and the dialysate collected. PTZ induces the depolarization of neurons and can cause seizures at high doses; therefore, a spike in extracellular glutamate is expected. Indeed, PTZ administration produced a large spike in both glutamate (E) and its analogue aspartic acid (D) within 5 minutes of administration **(Fig 3B, arrows)** with little to no changes in other amino acids demonstrating successful microdialysis and measurement of glutamate in the murine frontal cortex.

Extracellular concentrations of amino acids were then measured following infection with *Toxoplasma* and compared to values before infection. Glutamate concentrations remain low and constant during the first 21 days however, consistent with our observations of astrocyte and neuronal morphologies, a significant increase is observed at and after 28 days and continues to increase over the course of infection **(Fig 3C)**(one way ANOVA; p = 0.0003).

The dysregulation of glutamate can occur through a number of mechanisms. As we have documented extreme sustained swelling of astrocytes, one of these mechanisms could be via the opening of volume regulated anion channels (VRAC) and release of intracellular glutamate. However, measurement of aspartate and glutamine, both of which would also be released simultaneously through these channels, did not exhibit the same pattern of increasing concentrations and indeed remained relatively constant throughout infection suggesting that the non-specific dumping of amino acids as would occur through VRACs is a less likely mechanism. Further, glutamine is the most prevalent amino acid in the CNS and its extracellular concentration is not directly regulated by GLT-1, GS or GLAST. Although aspartate is co-transported with glutamate, its extracellular levels can be maintained independently of GLT-1 and GLAST. Both glutamine and aspartate exhibit non significant changes during the course of infection indicating that changes observed in glutamate are not due to an experimental artifact (p = 0.2023, p = 0.2227 respectively). The remainder of the non-essential amino acids mostly exhibited a U-shaped curve, with extracellular concentrations sharply increasing in early acute infection, then decreasing back to naïve levels between days 10 and 28 only to increase again during the chronic stage **(Figs 3D and S2)**. Using a one-way ANOVA, significant kinetic changes in extracellular levels of Tryptophan (p = 0.0071), Histidine (p = 0.0403), Lysine (p = 0.0243), Phenylalanine (p = 0.0229), Proline (p = 0.0168), Serine (p = 0.04), Threonine (p = 0.0052) and Tyrosine (p = 0.0149) were measured during the course of infection. A Dunnett’s post-test was performed for all timepoints against naïve concentrations and significance shown as asterisks (**Figs 3C, 3D and S2**). One notable exception is arginine, which continuously decreases during the course of infection **(Fig 3D)** (p<0.0001). Arginine is an important substrate for anti-microbial factors including nitric oxide, which is readily made during infection with *Toxoplasma*. Essential amino acids show a similar U-shaped response **(S2)**, which suggest a global disruption of brain metabolism during the course of infection. To summarize, measurement of ECS amino acids over the course of CNS *Toxoplasma* infection reveals significant time dependent changes in concentrations. However, although well buffered during acute systemic infection, we measure a specific and sustained increase in glutamate in the brain during chronic infection. These data point to the downregulation of GLT-1 as a possible cause of glutamate-induced excitotoxicity during infection.

### Ceftriaxone does not alter the immune response to *Toxoplasma* infection

**Fig 4:**
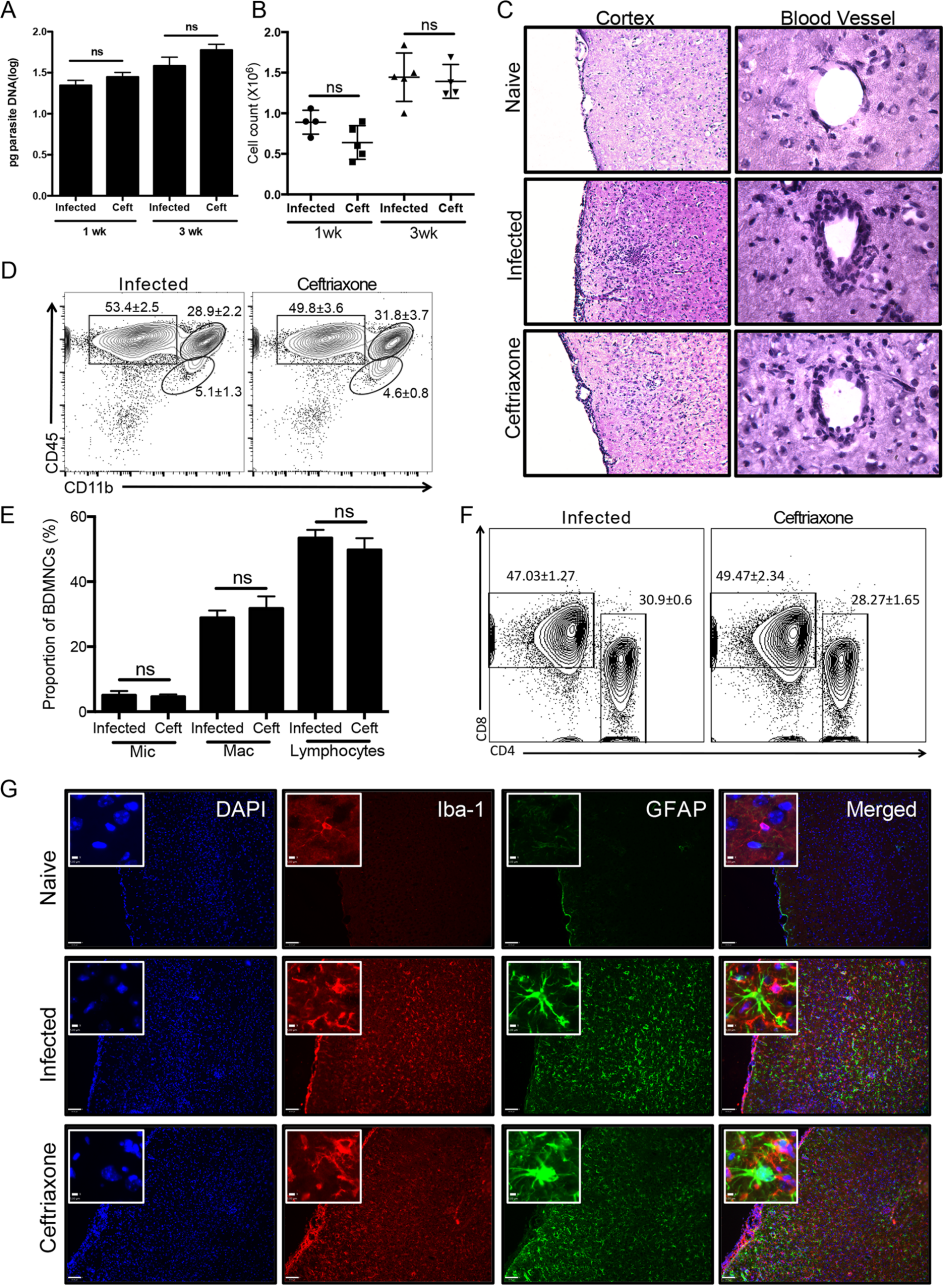
Ceftriaxone does not alter the immune response to *Toxoplasma* infection. Treatment of infected mice with ceftriaxone was conducted for one or three weeks starting at 5 weeks post-infection and compared to naïve and untreated infected mice. A) Parasite burden was quantified by RT-PCR of forebrain DNA extracted at one week post treatment (6 weeks post infection) from infected (n = 4) and ceftriaxone (n = 5) treated animals (Student’s t-test: infected vs. ceftriaxone p = 0.2730) and at three weeks post treatment (8 weeks post infection)(Student’s t-test: infected (n = 5) vs. ceftriaxone (n = 4) p = 0.2902). B) Brain mononuclear cells (BMNC) were extracted following one week of treatment from infected (n = 4) and ceftriaxone (n = 5) treated brains and counted (Student’s t-test: infected vs. ceftriaxone p = 0.0826) and after 3 weeks of ceftriaxone treatment (Student’s t-test: infected (n = 5) vs. ceftriaxone (n = 4) p = 0.7794). C) Hematoxylin and eosin staining was performed on 3 week treated mice and images of the frontal cortex (10X) and blood vessels (25X) within the frontal cortex were taken. D) Flow cytometry of BMNC reveals the lymphocyte (CD45^+^CD11b^-^), macrophage (CD45^+^CD11b^hi^) and microglial (CD45^int^CD11b^int^) populations following 3 weeks of treatment with ceftriaxone. E) Quantification of the proportions of BMNC between infected (n = 5) and ceftriaxone (n = 4) treated mice for microglia (p = 0.7804), macrophages (p = 0.5013) and lymphocytes (p = 0.4164). F) Flow cytometry of BMNC for CD4 and CD8 (Student’s t-test: CD4 infected vs. ceftriaxone p = 0.2075; CD8 infected vs. ceftriaxone p = 0.4122). G) Immunohistochemistry for the microglial marker Iba-1 and the astrocytic marker GFAP was performed on 12μm frozen sections from the frontal cortex (scale bar: 80μm); inserts, high magnification images of cell morphology (scale bar: 5μm).

To determine the degree to which GLT-1 is responsible for the range of neurological pathologies during *Toxoplasma* infection we tested the ability of the β-lactam antibiotic, ceftriaxone to manipulate the expression of GLT-1 in vivo. There are now a number of pharmacological agents that have been documented to increase GLT-1[55–57], however ceftriaxone is the most well characterized and has been tested in mouse models of ALS [58] and is neuroprotective in a variety of CNS injuries [59–61]. As with many antibiotics, ceftriaxone has also been reported to have anti-inflammatory properties [62–64]. To test the effects of ceftriaxone on *Toxoplasma* infection, we treated mice starting at 5 weeks post infection and sacrificed at either 6 weeks (1 week of treatment) or 8 weeks (3 weeks of treatment) post infection and compared inflammation and parasite burden to untreated infected control mice. There was no indication that ceftriaxone inhibited parasite growth or survival, with treated and untreated mice exhibiting no significant difference in parasite burden after one or three weeks of treatment (Student’s t-test: infected vs. ceftriaxone wk1: p = 0.2522; wk3: p = 0.2024) (**Fig 4A**). In addition, growth of tachyzoites *in vitro* was unaffected by ceftriaxone treatment (**S3 Fig**). No overt differences were noted between the inflammatory infiltrates in the meninges and in the perivascular space of the prefrontal cortex between mice treated with ceftriaxone and those of untreated mice **(Fig 4C)**. As a more quantitative measure of inflammation, brain mononuclear cell extraction was performed and immune cells counted; these typically include T-cells, macrophages and resident microglia. Mice treated with ceftriaxone exhibited a trend for a decrease in the number of total immune cells after 1 week of treatment; however this never reached statistical significance (Student’s t-test: infected vs. ceftriaxone p = 0.0826) and this trend was not maintained after three weeks of treatment (p = 0.7794) **(Fig 4B)**. Furthermore, flow cytometry analysis to quantify the proportions of resident microglia and infiltrating macrophages, CD8^+^ and CD4^+^ T-cells, showed that ceftriaxone did not alter the composition of immune cells within the brain **(Fig 4D–4F)**. This indicates that treatment with ceftriaxone does not impair or enhance immune cell infiltration in the CNS. Finally, to test if ceftriaxone inhibited the global astrocyte and microglial activation typically seen during infection, brain slices were probed with the microglial marker Iba-1 and antibodies to the astrocyte-specific filamentous protein, GFAP. Both treated and untreated infected mice exhibited increased microglial and astrocytic activation consistent and normal for *Toxoplasma* infection **(Fig 4G)**. Thus, although we cannot rule out potential anti-inflammatory effects that we have not measured, we conclude that administration of ceftriaxone induces minimal alteration of the immune response to *Toxoplasma* and does not lead to changes in parasite burden.

### Ceftriaxone increases GLT-1 and prevents infection-induced glutamate dysregulation

**Fig 5:**
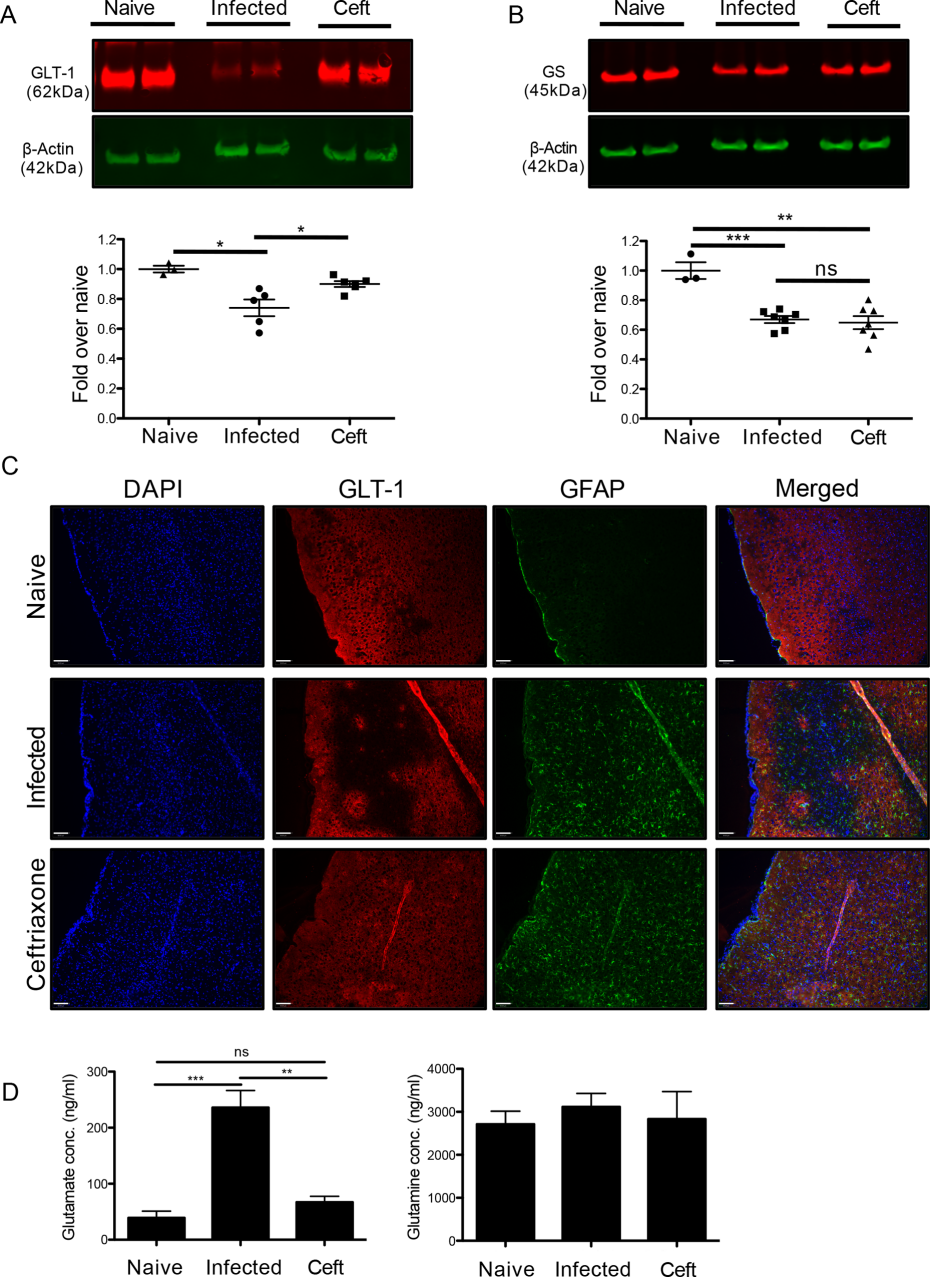
Ceftriaxone specifically rescues the glutamate transporter GLT-1. Treatment of infected mice with ceftriaxone was conducted for one week starting at 5 weeks post-infection and compared to naïve and untreated infected mice. Western blot of whole forebrain lysates for A) GLT-1 and B) GS on naïve (n = 3), infected (n = 5) and ceftriaxone treated (GLT-1 n = 5; GS n = 7) animals (Student’s t-test: GLT-1 naive vs. infected: p = 0.0144; GLT-1 infected vs. ceftriaxone: p = 0.0172; GS naïve vs. infected: p = 0.0002; GS infected vs. ceftriaxone: ns). C) Immunohistochemistry for GLT-1 and GFAP on 12μm thick frontal cortex sections (scale bar: 80μm). D) Glutamate and glutamine concentrations in the extracellular space of naïve (n = 4), infected (n = 6) and ceftriaxone (n = 3) treated mice as measured by microdialysis and LCMS (Student’s t-test: naive vs. infected: p = 0.0003; infected vs. ceftriaxone: p = 0.0072).

To determine if ceftriaxone could rescue GLT-1 expression in *Toxoplasma* infected mice, chronically infected C57BL/6 mice were treated, as before, with 200mg/kg i.p daily for one week starting at 5 weeks post infection. At 6 weeks, brains were harvested to measure protein expression of GLT-1. Mice infected with *Toxoplasma* exhibited a significant decrease in GLT-1 and GS protein compared to naïve mice **(Fig 5A and 5B)**. These data corroborate the decrease in GS and GLT-1 transcripts over the course of infection **(Fig 1B)**. When treated with ceftriaxone for one week, GLT-1 protein, but not GS, is partially rescued **(Fig 5A and 5B)**, supporting the specificity of ceftriaxone in regulating GLT-1 expression [58]. Immunohistochemistry for GLT-1 on serial sagittal sections reveals large areas negative for GLT-1 in the pre-frontal cortex of infected mice. Such patches were not associated with areas of cysts or individual parasites, as seen by DAPI staining or anti-*Toxoplasma* staining (**S4 Fig**). Co-staining with GFAP is consistent with downregulation of the transporter rather than astrocyte death. Following treatment with ceftriaxone, these large GLT-1-negative patches are no longer observed in the infected tissue **(Fig 5C)**. Loss of GLT-1 is still observed in the more resistant BALB/c strain **(S5 Fig)** suggesting that this is a common mechanism between mouse strains. Finally, to test whether the rescue of GLT-1 expression could restore extracellular concentrations of glutamate during infection, microdialysis was conducted on infected mice and infected mice treated with ceftriaxone. Measurement of ECS concentrations of glutamate revealed that mice treated with ceftriaxone have glutamate concentrations that significantly decreased from infected, untreated mice and were no different from naïve uninfected mice (naïve (n = 4) vs infected (n = 6): p = 0.0003; infected vs ceftriaxone (n = 3) p = 0.0072) **(Fig 5D)**. Ceftriaxone treatment did not affect ECS concentrations of glutamine further supporting the specificity of ceftriaxone action (**Fig 5D**). Thus, treatment of infected mice with ceftriaxone is able to restore GLT-1 expression and reduce ECS glutamate from pathological to normal concentrations in the infected frontal cortex.

### Ceftriaxone is neuroprotective during infection with *Toxoplasma*

**Fig 6:**
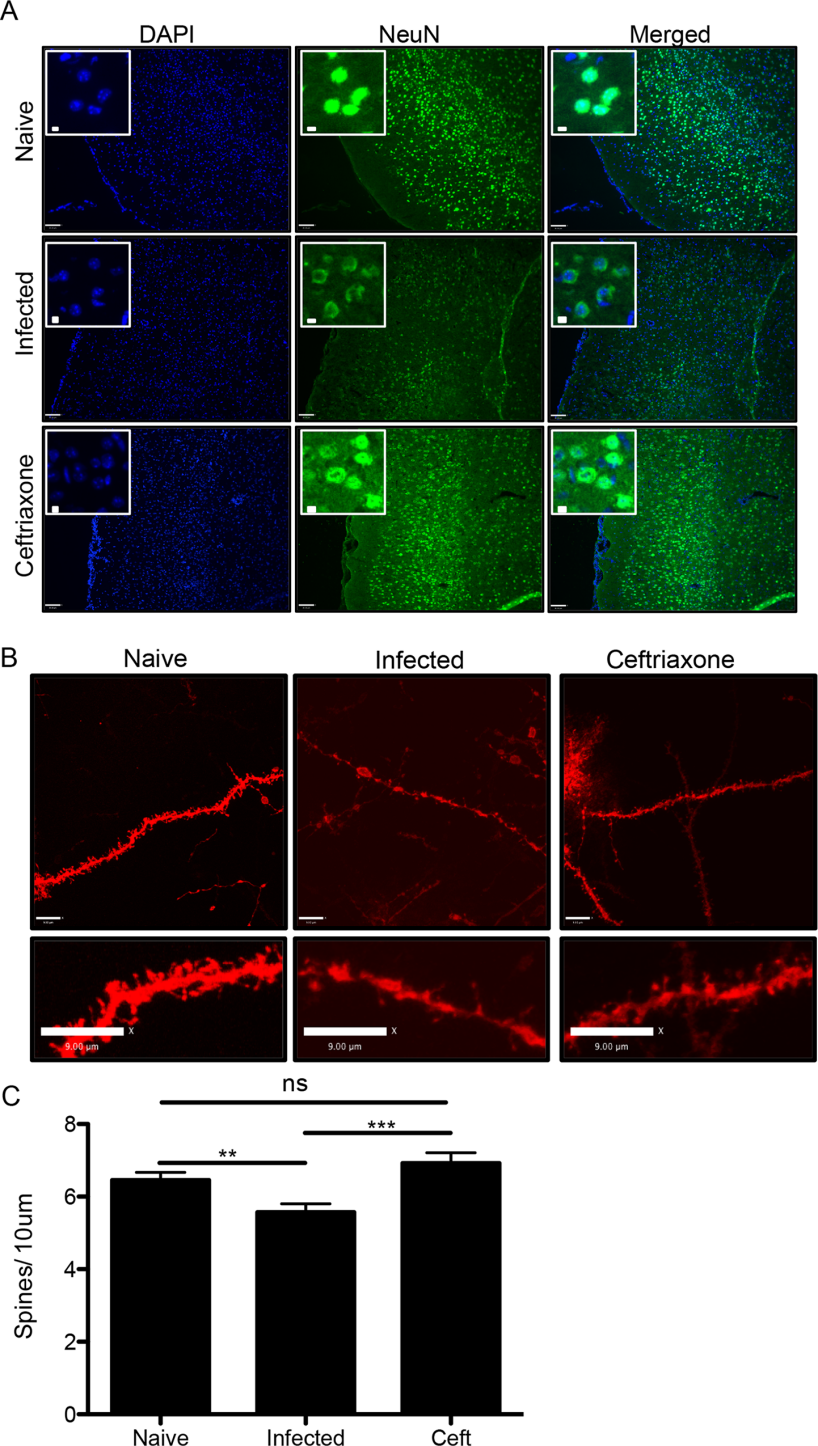
Ceftriaxone is neuroprotective during infection with *Toxoplasma*. Treatment of infected mice with ceftriaxone was conducted for one week starting at 5 weeks post-infection and compared to naïve and untreated infected mice. A minimum of 3 mice were used in each group and the experiments were repeated at least once. A) Immunohistochemistry for NeuN on 12μm thick frontal cortex frozen slices (scale bar: 80μm). Inserts: high magnification of NeuN immunohistochemistry (scale bar: 5μm) B) and C) DiI labeling of dendritic spines quantified in naïve, infected and ceftriaxone treated animals (Student’s t-test; infected vs ceftriaxone: p = 0.0004) (scale bars: 9μm).

The elevated levels of ECS glutamate observed during infection offer an explanation for the significant changes in neuronal morphology. To assess the protective effects of ceftriaxone on neuronal health during chronic infection, immunohistochemistry was performed for the neuronal nucleic marker NeuN. A marked decrease in staining intensity was observed in chronically infected mice when compared to uninfected naïve control mice. Some areas of the cortex were devoid of NeuN staining further supporting infection-induced damage to neurons **(Fig 6A)**. Mice treated with ceftriaxone for one week did not exhibit reduced NeuN staining in any cortical areas **(Fig 6A)**. Higher magnification confocal imaging reveal significant differences in the pattern of NeuN staining between naïve and infected animals. While NeuN is strongly positive in the nucleus of naïve neurons, it appears cytoplasmic and diffuse in the infected animals. Treatment with ceftriaxone protects the loss of nuclear NeuN **(Fig 6A)**.

To test if ceftriaxone can protect against synaptic loss in the frontal cortex, dendritic spine labeling was performed. As before, infection with *Toxoplasma* significantly reduces spine density in six week infected C57BL/6 mice compared to naïve (infected = 5.573±0.2288 spines per 10 μm and naive = 6.724±0.2572 spines per 10 μm; p = 0.0052) **(Fig 6B and 6C)**. In contrast, ceftriaxone treated infected mice did not exhibit any significant change in spine density when compared to naïve (ceftriaxone = 6.922±0.2904 spines per 10 μm and naive = 6.724±0.2572 spines per 10 μm; p = 0.2026) **(Fig 6B and 6C)**, and have significantly more spines than untreated infected mice (ceftriaxone = 6.922±0.2904 spines per 10 μm and infected = 5.573±0.2288 spines per 10 μm; p = 0.0004) **(Fig 6B and 6C)**. These data further demonstrate that infection with *Toxoplasma* is damaging to neurons and that treatment with ceftriaxone is effective in restoring such damage.

### Infection with *Toxoplasma* disrupts neuronal networks and changes behavior

**Fig 7:**
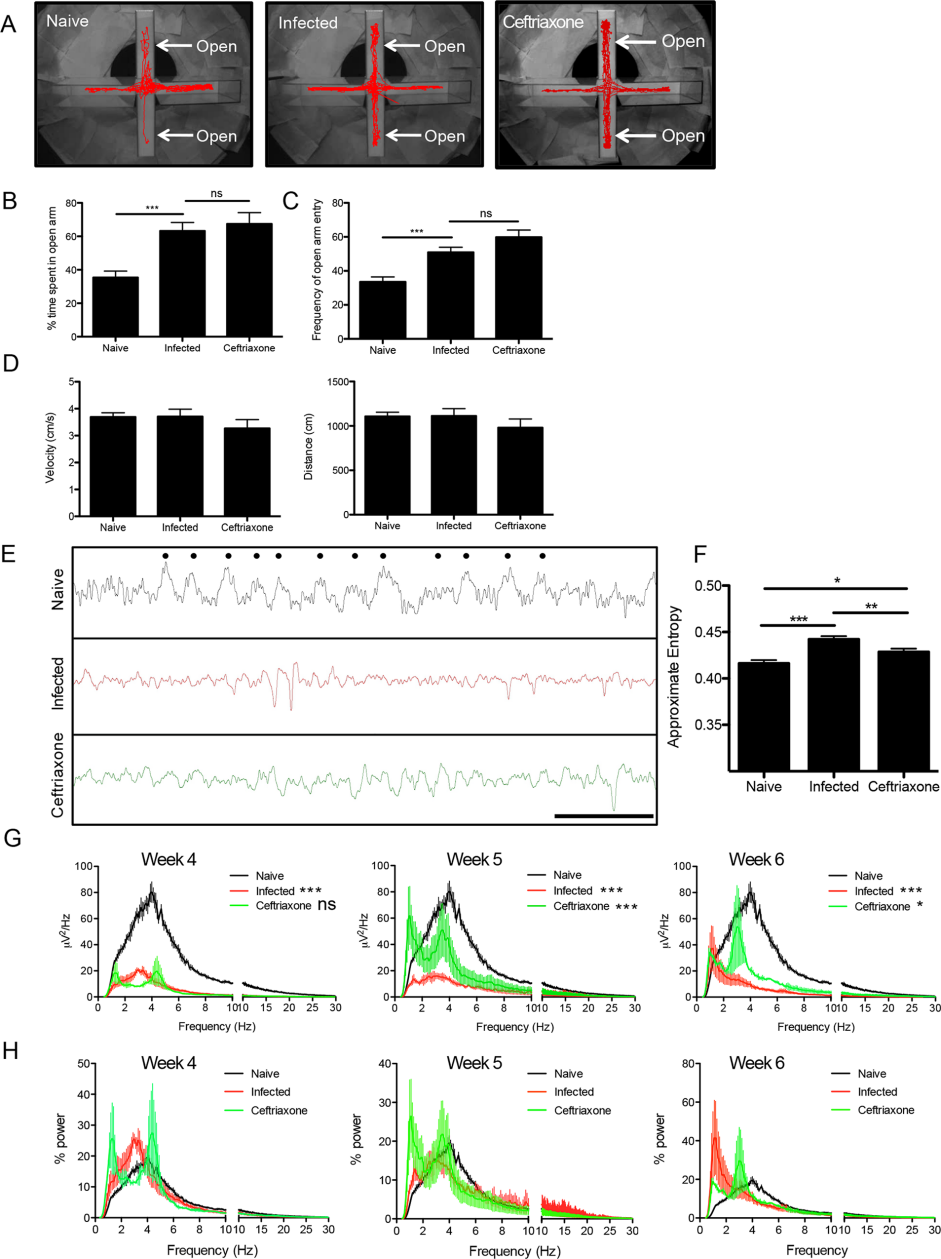
Infection with *Toxoplasma* disrupts neuronal networks and changes behavior. A)-D) Naïve (n = 13), 6 week infected (n = 11) and ceftriaxone treated (n = 11) mice were placed in the center of an elevated plus maze and recorded for 5 minutes. B) percentage, C) frequency of time spent in the open arm of the maze and D) velocity and total distance traveled was measured between naive and infected animals. E) EEG raw traces for naïve, 6 week infected and ceftriaxone treated animals (scale bar: 1sec; dots illustrate the peak of one full rhythmic cycle). F) Approximate entropies, Student’s t-test (naïve vs infected, p<0.0001; infected vs ceftriaxone, p = 0.0057; naïve vs ceftriaxone, p = 0.0138) G) EEG power density spectrum for naïve, 4, 5 and 6-week infected and ceftriaxone treated animals. A minimum of 3 animals were used for each group and the experiments repeated twice. Asterisks represented are Bonferonni’s multiple comparison test (infected vs naive) and (ceftriaxone vs infected) H) EEG percent power calculated from power density spectral analysis in G.

Our data are indicative of a global dysregulation of the main excitatory neurotransmitter in the brain, which could translate to functional and behavioral abnormalities in infected mice. To test whether changes in behavior are induced by chronic *Toxoplasma* infection, and if such changes are dependent on glutamate dysregulation, mice were subjected to the elevated plus maze anxiety test [13, 14, 65] **(Fig 7A)**. Following six weeks of infection, the time mice spent in the open arm of the elevated maze was assessed and compared to uninfected naïve, and ceftriaxone treated mice. Consistent with experiments conducted in rats [14] and mice [13], untreated infected animals placed on an elevated plus maze spent significantly more time in the open arms of the maze (p = 0.0002) **(Fig 7B)** and entered the open arms more frequently (p = 0.0005) **(Fig 7C)**. The observed increase in open arm activity is not due to an increase in the general activity of infected mice as there were no differences in the distance travelled or velocity compared to naïve mice (p = 0.9569 and p = 0.9561 respectively) **(Fig 7D)**. More time spent in the open arms suggest less anxiety. These data support similar studies [13, 14] and point to parasite-induced dysregulation of anxiety behavior. Although ceftriaxone treatment provided significant rescue of concentrations of glutamate and neuronal morphology, one week of treatment failed to modify infection-induced changes in behavior (**Fig 7A–7D**).

A more direct and functional read out of glutamate regulation is the measurement of neuronal activity. If glutamate is dysregulated on a global scale, then we would expect to see abnormalities in the collective firing of neurons. To test if the changes in behavior are paralleled by changes in normal electrical activity of the brain, resting state EEGs were performed on uninfected, chronically infected and ceftriaxone treated mice. The EEG electrodes, placed in the frontal cortex, measured brain activity for 24-hour periods allowing for a detailed analysis of EEG waveform and power that reflects strength and synchronization of neuronal circuits. Due to the technically challenging aspects of this experiment, ceftriaxone treatment began at 3 weeks post infection and continued throughout the experiment with analysis conducted at 4, 5 and 6 weeks post infection. Analysis of immune responses and parasite burden following this altered treatment regime did not reveal any significant difference in parasite burden or immune infiltrate between untreated and treated mice (**S6**). Following six weeks post infection; cortical EEG rhythms clearly seen in naïve mice (**Fig 7E, top**) are disrupted in infected mice with a reduction in amplitude and frequency **(Fig 7E, middle)** suggesting a reduction or perturbation in synchronous neuronal firing. The degree of pattern in an EEG epoch can be quantitatively measured using approximate entropy; this technique can quantify the level of unpredictability in a time series, with larger values representing less regularity. In line with ceftriaxone’s neuroprotective qualities and its rescue of dendritic spine density, treatment partially rescues this EEG measure (naïve vs infected, p<0.0001; infected vs ceftriaxone, p = 0.0057; naïve vs ceftriaxone, p = 0.0138) **(Fig 7E and 7F)**. EEG power over the physiological frequency range was then quantified as a measure of neuronal synchrony and normal brain function. A dramatic loss in EEG power over all frequencies is observed at 4, 5 and 6 weeks post infection compared to naïve (One way ANOVA: p<0.0001 Bonferroni’s post test: p<0.001 between naïve and infected for week 4, 5 and 6) **(Fig 7G and 7H)**. This loss in EEG power at week 6 is accompanied by a reduction in characteristic frequency so that lower frequencies become dominant in the *Toxoplasma*-infected state. When ceftriaxone is administered for one week, there is no rescue of EEG power and defects remain across all frequencies (One way ANOVA: week 4, p<0.0001 Bonferroni’s post test not significant between infected vs ceftriaxone). However, continued treatment with ceftriaxone partially rescues EEG power at week 5 (One way ANOVA: week 5, p<0.0001 Bonferroni’s post test (infected vs ceftriaxone: p<0.001) and week 6 post-infection (One way ANOVA: week 6, p<0.0001 Bonferroni’s post test (infected vs ceftriaxone: p<0.05) **(Fig 7G)**. Overall the ceftriaxone-treated group demonstrated EEG power spectra at weeks 5 and 6 that were intermediate between naïve and infected, untreated mice **(Fig 7G, weeks 5 and 6).** Although the ceftriaxone treated group is particularly variable, no significant differences in power allocation were observed among all 3 groups across weeks 4, 5 and 6 weeks post infection (One way ANOVA) **(Fig 7H)**. These data demonstrate that infection with *Toxoplasma* decreases electrical activity and neuronal connectivity within the frontal cortex that can be partially rescued by prolonged ceftriaxone treatment.

## Discussion

In this report, we set out to characterize CNS pathology and its effect on normal brain function during *Toxoplasma* infection. We have demonstrated that during chronic infection, when the parasite and inflammation have reached equilibrium, there is sustained neuronal pathology that manifests as an alteration in neuronal connectivity and output. We further show that this pathology is at least in part dependent on the dysregulation of the primary neurotransmitter glutamate, and can be rescued by ceftriaxone treatment and upregulation of the glutamate transporter GLT-1.

These data suggest that although infected mice are outwardly indistinguishable from their naïve counterparts, chronic CNS pathology is present. Microdialysis experiments reveal dynamic changes in ECS amino acids during the course of infection, including a robust increase in ECS glutamate concentration. The specificity of glutamate synthesis and regulation by astrocytes leaves the CNS vulnerable to excitotoxicity by lack of compensatory mechanisms to regulate glutamate. This evidently occurs during CNS injuries, infections and neurodegenerative diseases, as several are characterized by an increase in CNS extracellular glutamate [33, 35–41]. ECS glutamate can reach excitotoxic levels either by decreased glutamate uptake, increased secretion during neuronal transmission, or both. During *Toxoplasma* infection, a decrease in expression of GLT-1 leads to an increase in ECS glutamate which may be exacerbated by increased glutamate release through the glutamate/cysteine antiporter xc^-^ system expressed by microglia and other cells in the CNS during inflammatory events [66–69]. Our data suggest that restoring glutamate regulation by increasing GLT-1 levels returns glutamate concentrations to that of uninfected controls and rescues much of the neuronal pathology observed. There are limitations to pharmacologically increasing GLT-1, as the specificity of the drug will always be hard to verify. Ceftriaxone has the potential to modulate the immune system, however, we recorded no differences in immune cell numbers or parasite burden suggesting that any anti-inflammatory effects of ceftriaxone were limited and not enough to prevent parasite control. Furthermore, we not only demonstrate specific upregulation of GLT-1 by ceftriaxone, but also restoration of extracellular glutamate to baseline levels. Thus, although there may be multiple actions for ceftriaxone, our data suggest that the dominant effect is restoration of extracellular glutamate.

Cell-cell communication via gap junctions (astrocytes) and synapses (neurons) facilitate CNS function while intimate feedback loops connect neuronal circuits. Therefore, our discovery of a significant disruption in the regulation of the glutamate pathway could predict changes in other neurotransmitters. Indeed, while contradictory reports exist for the ability of *Toxoplasma* to manipulate dopamine [70–72], recent work investigating GAD67 localization at the presynaptic terminal demonstrates disruption in GABA signaling and a decrease in seizure threshold [73]. Decreased inhibition and increased glutamatergic signaling is a clear pathway to seizures and occurs in patients with Toxoplasmic encephalitis [74]. In addition, here we demonstrate that network connectivity as measured by EEG is significantly reduced in a chronic and controlled infection. Resting state EEG abnormalities have been detected in a variety of neurodevelopmental, psychiatric and neurodegenerative disorders, such as autism spectrum disorders [71], bipolar disorder [75], schizophrenia [75], Huntington’s disease [76] and Alzheimer’s disease [77]. In particular, in a variety of these studies, reduced EEG power indicating reduced functional connectivity correlates with neurological and cognitive impairment. Thus, reductions in EEG power as observed with *Toxoplasma* infection, in the frontal lobe in particular are likely to correlate with frontal lobe cognitive dysfunction. Such cognitive deficits have been widely reported in the human population [9, 78–81]. Experiments in mice have demonstrated an effect of infection on olfaction and behavior towards bobcat urine [15]. The mechanism, of what must be a highly specific modulation of neuronal activity has recently been described as epigenetic control of the arginine vasopressin promoter of the amygdala [82]. However, such specific alterations are less likely to lead to the wide range of human disorders correlated with infection. Other more recent studies have described widespread changes in neurons [83], pathology, and behavior [47, 50] including MRI analysis and loss of the synaptic marker PSD95, supporting the concept that connectivity and neuronal output is changed [83]. Our study now adds to this by demonstrating a reduction in brain wave activity, dendritic spine density and disruption of neuronal synaptic markers.

Our data suggest that the alterations in neuronal morphology and network activity is linked to downregulation of GLT-1, as increasing GLT-1 expression via ceftriaxone treatment rescues the majority of this pathology. However, recovery of GLT-1 expression and glutamate levels failed to rescue the altered risk seeking behavior as measured by the elevated plus maze. This could indicate that this particular behavior is independent of glutamate dysregulation. Perhaps changes in other neurotransmitter systems—such as dopamine or GABA, play a larger role in anxiety behavior which would not be restored by administration of ceftriaxone. Failure to rescue risk-seeking behavior could also suggest that ceftriaxone restores synapses to pre-infection numbers but that the synaptic connections are remodeled. Future studies including a battery of behavioral tests may be needed to determine the effects of glutamate dysregulation on behavior during *T*. *gondii* infection.

Studies using mice heterozygous for GLT-1 in which protein expression is 45–60% less than that of wild type exhibit enhanced protection during ischemia [34] suggesting loss of GLT-1 does not always lead to severe neurological disease. The caveats to this are the unknown compensatory mechanisms that may be present when genetically manipulating GLT-1, however the ability to buffer the system may depend on the context in which GLT-1 loss occurs, in our case chronic infection, a less pathological initial insult than ischemia but continuous. It is also interesting that GLT-1 heterozygous mice exhibit behavioral abnormalities similar to those seen during *Toxoplasma* infection including lower anxiety responses [84], which may simply reflect the importance of glutamate control in this specific network.

A primary area for future consideration is what initiates the downregulation of GLT-1 during chronic *Toxoplasma* infection. Surprisingly, considering the importance of this transporter to maintaining glutamate homeostasis, there is little understanding of the mechanisms that govern GLT-1 expression. Reports have documented the ability for nitric oxide, a molecule that is certainly expressed during chronic *Toxoplasma* infection (66, 67), to induce GLT-1 downregulation [85, 86]. Although an increase in glutamate concentration is not apparent until chronic time points, modification of GLT-1 transcripts are initiated much earlier when systemic inflammatory responses will be detectable at the blood brain barrier interface. As there seems to be a delay in changes in GLT-1 protein expression and glutamate concentrations compared to transcription, it is possible that there is differential regulation of GLT-1 splice variants [87, 88]. Also, immediate and subtle changes apparent during acute infection may not materialize to changes at the protein level or extracellular glutamate concentrations for some time. As GLT-1 is abundant in the brain, and we see loss occurring in patches, it seems likely that glutamate uptake is only impaired at a certain ‘threshold’ level of GLT-1 protein deficiency.

GLT-1 downregulation occurs in many disease states [33–40] and is therefore not unique to *Toxoplasma* infection. However, the observation that GLT-1 loss occurs in patches suggests a more focal source of downregulation than a circulating factor or general inflammation. Although no direct correlation with parasite location was found, this is hard to rule out as a tissue slice represents such a small area in time and space. Preliminary experiments using cultures of primary astrocytes do suggest that direct parasite invasion could contribute to GLT-1 inhibition. As GLT-1 expression is reported to be regulated via NF-kappaB, a pathway that is a target of parasite inhibition, there is a mechanistic avenue for this [89][90, 91]. Although direct parasite infection and manipulation of host cell machinery is clearly a favored hypothesis, recent work suggests that the frequency of astrocyte infection *in vivo* is extremely low [92]. Whether those few infected astrocytes would be sufficient to trigger such a substantial downregulation of GLT-1 seems unlikely and would indicate a very susceptible system. The more likely hypothesis is that infection within neurons leads to a change in neuronal/astrocyte communication [89]. Astrocytic expression of GLT-1 is dependent on the presence of neurons and continuous feedback between these cells regulates its expression [93, 94]. New detailed analysis of cyst biology reveals a far more active and variable bradyzoite population than previously appreciated [45], giving the cyst greater potential to alter the host neuron and those cells in its vicinity. Considering GLT-1 downregulation is apparent in many neurological diseases without the presence of parasites, it is unlikely that direct parasite interaction is the only mechanism that downregulates GLT-1 during infection. Instead, additional inflammatory mediators that are a constant and necessary part of chronic *Toxoplasma* infection in the brain likely also play a role. Future studies will address the degree to which *Toxoplasma* can manipulate GLT-1 expression versus the innate astrocytic response to the ongoing inflammatory event. Clearly, the prevalence of *Toxoplasma* infection is far greater than the neurological diseases that have been linked to it and so interpreting these data as altered neuropsychology and behavior in *Toxoplasma*-infected people should be avoided, however, it is increasingly recognized that the nervous and immune systems are intimately connected. The finding that chronic *Toxoplasma* infection in the brain induces significant disruption of neurotransmitters may support the theory that such an infection could trigger neurological disease in those already genetically predisposed.

## Methods

### Animals

All animal research has been done in accordance to the Animal Welfare Act. All protocols were approved by the Institutional Animal Care and Use Committee (IACUC) of the University of California, Riverside. All surgery was performed under isofluorane anesthesia, and all efforts were made to minimize suffering. Female C57BL/6 and BALB/c mice were obtained from Jackson Laboratories and maintained in a pathogen free environment under IACUC established protocols at the University of California Riverside.

### Infections and treatments

Female C57BL/6 or BALB/c mice were infected intraperitoneally with 20 cysts of the Me49 strain in 200μl of sterile Na+/K+ balanced PBS. Ceftriaxone (TCI) was administered at 200mg/kg intraperitoneally for between 7 and 21 days starting at day 35-post infection (or on occasion day 21-post infection for EEG/microdialysis experiments) as specified. Care was taken in alternating injection side each day. Age and sex matched control animals were injected with saline and having received no antigen are termed “naïve” throughout. Saline injections were administered to infected, untreated animals to control for ceftriaxone treatment.

### Quantification of *T*.*gondii* burden

For in vivo analysis, half brains were homogenized and parasite burden was measured by amplifying the *T*. *gondii* B1 gene by real-time PCR as previously described [95]. For in vitro analysis human foreskin fibroblasts (HFF) were cultured in triplicate and infected at a MOI of 3:1. Uninvaded parasites were removed after 3hrs and cells were cultured for a further 24hrs. Media was removed and cells were cultured for a further 24hrs with fresh media containing various concentrations of ceftriaxone ranging from 0.1–1000μM. Infected cells were harvested and RT-PCR was conducted for the *Toxoplasma* B1 gene.

### Anesthesia and surgery

Mice were anesthetized in an induction box with 3.5% isofluorane and maintained with continuous administration of 2.5% isofluorane through a nose cone. All surgeries were performed prior to infection and mice allowed to recover for a minimum of 7 days before being infected.

#### Microdialysis

For microdialysis experiments, uninfected mice were placed in a stereotaxic frame and a 1.5 cm incision was made in order to expose the junction of the coronal and sagittal sutures (bregma). A burr hole was made with a 1mm drill bit 2.58mm rostral and 1.5mm lateral from bregma using a stereotaxic mouse atlas. A CMA 7 guide cannula (CMA P000137) was then lowered 0.5mm ventrally and held in place with glass ionomer cement (CMA-72-9168). Two additional holes were made on each lateral side of bregma using an anchor screw drill bit (CMA 8003264) to place 2 anchor screws (CMA 7431021) into the skull. The skull was then roughened to allow optimal ionomer bonding by using a 1mm drill bit and gently scraping the entire skull surface around the anchor screws and the guide cannula. Finally, a tether bolt (CMA 61–0037) was held in place while the ionomer cement was applied over the anchor screws, tether bolt and guide cannula. The skin was then gently lifted over the cement and the mouse was allowed to recover on a 38°C mat.

#### Electroencephalography

For electroencephalography (EEG) experiments, a burr hole was made as described above at the same stereotaxic coordinates. A twisted bipolar stainless steel bipolar electrode (Plastics One) was inserted 1mm under the dura mater and grounded to the dura. The EEG implant was held in place with glass ionomer cement.

### Electron microscopy

Female C57BL/6 mice infected with Me49 were sacrificed at 3, 6 and 12 weeks post infection and perfused intra-cardiacally with 2.5% gluteraldehyde, 4% paraformaldehyde (PFA) in 0.1M sodium cacodylate buffer. Brains were removed and post-fixed in the same buffer. Serial electron microscopy of the frontal cortex of the brain was performed by Renovo Neural inc, (Cleveland, Ohio). 6–10 Z stacks from different areas of the frontal cortex of naïve and infected mice (naïve n = 1; 3 weeks n = 3; 6 weeks n = 3; 12 weeks n = 2) were collected. Blood vessels ~5–6μm wide were selected (naïve n = 20; 3 weeks n = 75; 6 weeks n = 134; 12 weeks n = 82) and average astrocyte endfeet width was quantified by measuring perivascular astrocyte area and dividing by the blood vessel circumference using Fiji software. Significance was tested using Student’s t-test.

### Real Time PCR

Female C57BL/6 mice infected with Me49 were sacrificed at day 7, 14, 21, 28, 35, 42 and 56 post infection (n = 3 per timepoint), and perfused intra-cardically with sterile PBS. Brains were removed and homogenized in TRIzol (Ambion). RNA was extracted using the TRIzol/ chloroform method and concentrations of nucleic acids were determined on a nanodrop 2000. GLT-1 specific primers (forward 5′-ACCTTGCAATCCCTCTTCGG-3′ and reverse 5′-AGACCGGTACCAGGAGTGG-3′), GLAST specific primers (forward 5′-CTGGTAACCCGGAAGAACCC-3′ and reverse 5′-GGGGAGCACAAATCTGGTGA-3′), and Glutamine synthetase specific primers (forward 5′ -ACCCCTATGCGGTGACAGAA-3′ and reverse 5′-CGTCGCCTGTTTCGTTGAG-3′) for Real Time PCR were purchased from IDT's primer Quest. cDNA synthesis and Real-time PCR were performed using the Bioline One-step Kit with the CFX-96 real-time PCR Detection System (Bio-Rad). The reaction total was a 20μl mixture with 10μl SYBR Green/SensiFAST qPCR Master Mix (2x) and 400nM primer. The reaction conditions were as follows: 10 min at 45°C, followed by 2 min at 95°C and then 40 cycles of 5s at 95°C and 20s at 60°C. The GAPDH (Glyceraldehyde 3-phosphate dehydrogenase) forward primer (5′-AGGCCGGTGCTGAGTATGTC-3′) and reverse primer (5′-TGCCTGCTTCACCACCTTCT-3′) were used as an endogenous control. Quantified results represent the fold induction of target gene expression using the differential CT method. NTC, no-template control (reagent alone without template) was included in each assay to detect any possible contamination of the PCR reagents.

### Western blots

Brains from naïve and 6-week infected C57BL/6 mice were homogenized in protein lysis buffer with DTT and protease inhibitors using the Bullet Blender (Next Advance). Protein concentrations were determined using a BCA protein assay kit (Thermo Scientific). 2μg (for GLT-1) or 5μg (for GS) was denatured at 95°C for 5 minutes in 2μl 10% SDS, 5μl loading buffer, and topped off to 20μl with RIPA buffer. A 10% Tris-HCL gel (Bio-Rad), was loaded and run at 50–100V in running buffer. The gel was transferred to a nitrocellulose membrane using the semi-dry method in blotting buffer. The membrane was blocked for 1 hour in blocking buffer (5%w/vol BSA, 1X TBST), washed in TBST and incubated with primary antibodies (Rabbit anti-Glutamine Synthetase, Sigma at 1μg/ml; Rabbit anti-GLT-1, Thermo Scientific at 50ng/ml; Rabbit-anti VGlut-1, Invitrogen at 2.5μg/ml) overnight at 4°C. The membrane was then washed in TBST and incubated in secondary antibodies (0.4μg/ml; eBiosciences) and imaged on a fluorescent imager (Odyssey, LI-COR Biosciences).

### Immunohistochemistry

Six week infected mice were sacrificed and perfused intra-cardically with PBS followed by 4% PFA. A minimum of 3 biological replicates was used for each antibody combination and the experiment performed at least three times. Brains were extracted, post-fixed overnight in 4% PFA followed by a 3 day 30% sucrose equilibration. Brains were frozen at -80°C in optimal cutting temperature compound (OCT). 10–15μm cryostat sections were then blocked with 10% donkey serum in PBS for 1 hour at room temperature. Primary antibodies against GLT-1, GFAP, ß-III-Tubulin, Iba-1, NeuN, VGlut-1 and *Toxoplasma* (Rat anti-GFAP, Invitrogen used at a final concentration of 5μg/ml; Rabbit anti-GLT-1, Thermo Scientific at 44ng/ml; Rabbit-anti VGlut-1, Invitrogen at 2.5μg/ml; Chicken anti-ß-III-Tubulin, Chemicon at 0.8μg/ml; Anti-NeuN, Abcam at 7.3μg/ml; Anti-Iba-1, Abcam at 35μg/ml; Rabbit-Anti-*Toxoplasma*, Abcam at 50μg/ml) were incubated overnight at 4°C in 10% donkey serum and 0.5% Tween-20 in PBS. After several washes in PBST, the slices were incubated 3 hours at room temperature with Alexa Fluor fluorescent secondary antibodies (4μg/ml; eBiosciences). Slices were then washed and mounted with ProLong Gold (Molecular Probes).

#### ß-III tubulin quantification

Serial 10-micron coronal pre-frontal cortex sections stained for ß−III tubulin were imaged at 63X using a Zeiss 510 confocal microscope. 8 Micron thick Z-stacks were imaged and positive cells counted using Volocity image analysis software.

#### VGlut1 quantification

*VGlut-1 puncta quantification*: Serial 10-micron coronal pre-frontal cortex sections stained for VGlut-1 were imaged at 63X using a Zeiss 510 confocal microscope. 8 Micron thick Z-stacks were imaged and analyzed for puncta density using Volocity image analysis software. Western Blot analysis was performed as described above from whole brain lysates.

#### Nissl staining

40μm sections were cut and stained with Cresyl Violet Solution (IHCWORLD) as follows. Rehydrate slices in 1 min in 100% EtOH, 1 min in 95% EtOH, and 1 min in distilled water. Stain with Cresyl Violet Solution for 6 min at room temperature then rinse in distilled water. Differentiate stain in 95% EtOH for 2 min and dehydrate slices in 2 changes of 100% EtOH for 5 min each. The slices were then cleared in 2 changes of CitriSolv (Fisher) for 5 min each and mounted. Nissl stained sections were imaged under 400X total magnification and images of layer 2/3 of the prefrontal cortex were taken from serial sections. Neurons from naïve (n = 35 ROIs each 150μm by 200μm, 1982 neurons) and infected (n = 34 ROIs each 150μm by 200μm, 2073 neurons) sections were counted manually using strict morphological guidelines.

### Microdialysis

Surgery was performed as described above and mice allowed to recover for a minimum of 7 days prior to infection. Microdialysis was performed on uninfected (N) and day 3, 7, 10, 14, 21, 28, 35 and 42 post-infection of C57BL/6 mice with a minimum of 4 mice per group. Naïve samples were harvested prior to infection and throughout the course of infection to rule out changes in baseline concentrations due to the presence of implants or age. No significant change in baseline was recorded. In the case of ceftriaxone treatment microdialysis was started at 5 weeks post infection and measurements taken after 1 week of treatment (6 weeks post infection). Mice were placed in microdialysis cages which allowed free movement and access to food and water. 1mm CMA 7 probes (CMA 000082) were implanted through the guide cannula and hooked to a microdialysis syringe pump. Artificial cerebral spinal fluid (CMA P000151) was perfused at a flow rate of 0.6 μl/min and collected in a 1.5ml eppendorf tube. Mice were perfused for 5 hours prior to sample collection to allow for equilibration of the CNS. Samples were collected over a 3 hour period and placed on dry ice. Samples were sent to Sussex Research Laboratories for amino acid analysis via LC-MS. Data were analyzed using a one-way ANOVA followed by a Dunnett’s post test for each timepoint against naïve concentrations of each amino acid. Results reported as p-values for ANOVA and asterisks for the Dunnett’s multiple comparison tests.

### Dendritic spine analysis

6 week infected; infected and ceftriaxone treated and age matched naïve (uninfected) C57BL/6 mice were sacrificed and perfused intracardically with PBS followed by 4% PFA. Brains were extracted and post-fixed 2 hours in 4% PFA, and stored in PBS. 100μm coronal section were sliced using a vibratome and labeled using DiI coated tungsten particles administered through a gene gun. Dendritic spines in layer 2/3 of the prefrontal cortex were imaged using a Zeiss 510 confocal microscope at a magnification of 63X. 15μm Z-stacks were obtained from 5 slices and 3–4 brains per group. For each hemisphere, 5 images were taken and all spines within those images counted using Volocity 3D imager (Perkin Elmer), resulting in ~10 000 spines per condition.

### Electroencephalography recording and analysis

Mice were connected to the MP150 EEG recording module (Biopac Systems inc.) and EEG traces recorded for 24 hours at a sampling rate of 4000 samples/ second using the AcqKnowledge 4.4 software. Raw EEG traces were filtered using a digital FIR band pass filter between 0.8hz and 30Hz. A fast Fourier transform followed by a power spectrum density analysis was performed to measure power over the set frequency range of 8–10 30-minute traces for each experimental group. Percent power was calculated by dividing power at each frequency by total power (area under curve) and multiplying by 100. At least 3 mice per group were used and the experiment repeated twice. Data were analyzed using a one-way ANOVA followed by a Bonferroni’s multiple comparison’s test. For entropy analysis, 7 filtered 2min traces for each group were analyzed for approximate entropy using the following parameters: epoch: 2s; order (m): 2 and filtering level (r): 0.2. All analysis was performed on AcqKnowledge 4.4 software.

### Behavioral experiments

Naïve (n = 13), 6 week infected (n = 11) and 6 week infected and treated with ceftriaxone for 1 week (n = 11) C57Bl/6 mice were placed at the center of a standard elevated plus maze and recorded with an overhead camera for 5 minutes. Videos were analyzed blindly with EthoWatcher software; distance travelled and velocity was measured. Additionally, number of open arm entries and time spent in the open arms of the elevated plus maze was measured.

### Statistical analysis

Unless otherwise stated, Student’s t-test was used to determine significance between all parametric data. The equality of variance between means was calculated with the F-test using GraphPad Prism 6 software. A Dunnet’s post-test was used to test significance of amino acid sampling over the course of infection with naïve, uninfected samples taken throughout the time course. Bonferonni’s multiple comparison test was used to test significance of EEG power analysis.

## Supporting Information

S1 Fig Electron microscopy analysis of astrocytic endfoot width. C57Bl/6 mice were infected with *Toxoplasma* and brains harvested. Scanning serial electron microscopy images analyzed for astrocytic endfeet width (Fig 1). Micrographs pictured here without highlighted borders for clarity.
(TIF)

S2 Fig Infection with *Toxoplasma* induces dynamic changes in essential amino acids. Microdialysis was performed over the course of *Toxoplasma* infection taking measurements prior to (N) and after infection as indicated (n = 13 biological replicates (3 prior to infection; 2 for each time point thereafter)). LC-MS analysis on microdialysis samples over the course of infection. A one-way ANOVA: Tryptophan (p = 0.0071), Histidine (p = 0.0403), Lysine (p = 0.0243), Phenylalanine (p = 0.0229) and Threonine (p = 0.0052). A Dunnett’s post-test was performed for all timepoints against naïve concentrations and significance shown as asterisks. Amino acids not listed did not change significantly.
(TIF)

S3 Fig Ceftriaxone does not inhibit *Toxoplasma gondii* replication. Human foreskin fibroblasts (HFF) were cultured in triplicate and infected with A), C) and D) Prunigund (type II strain parasites) or B) RH (type I parasites) at a MOI of 3:1. Uninvaded parasites were removed after 3hrs, cells were cultured for a further 24hrs. Media was removed and cells were cultured for a further 24hrs with fresh media containing various concentrations of ceftriaxone (0.1–1000μM). A) Infected cells were harvested and RT-PCR was conducted for the *Toxoplasma* B1 gene. B)-D) cells histologically stained and percentage of cells infected and number of parasites per infected cell quantified from 3 infected wells per group.
(TIF)

S4 Fig GLT-1 loss is not directly related to parasite location. Brain sections from 6 week Me49 infected C57Bl/6 mice were immunohistochemically stained as described in methods.
(TIF)

S5 Fig Infection with *Toxoplasma* induces a loss in GLT-1 in the resistant BALB/c mouse. Western blot using protein derived from whole forebrain naïve (n = 3) and infected (n = 3) BALB/c mice were conducted and quantified for GLT-1 (Student’s t-test: p = 0.0081).
(TIF)

S6 Fig Treatment with ceftriaxone at three weeks post-infection does not alter the immune response to *T*. *gondii*. Age matched female C57Bl/6 mice were infected with *T*. *gondii*. At 3 weeks post-infection a cohort were treated with ceftriaxone (n = 4) and compared to uninfected naïve control mice (n = 4). A) Parasite burden was quantified using RT-PCR and B) total immune cell infiltration and phenotype were quantified using cell counts and flow cytometry. Using Student’s t-test no significant differences were measured.
(TIF)
