# Supplementary figures and images for: GLT-1-Dependent Disruption of CNS Glutamate Homeostasis and Neuronal Function by the Protozoan Parasite Toxoplasma gondii
Source: PLoS Pathog. 2016 Jun 9;12(6):e1005643. doi: 10.1371/journal.ppat.1005643 (PMC4900626; doi:10.1371/journal.ppat.1005643)

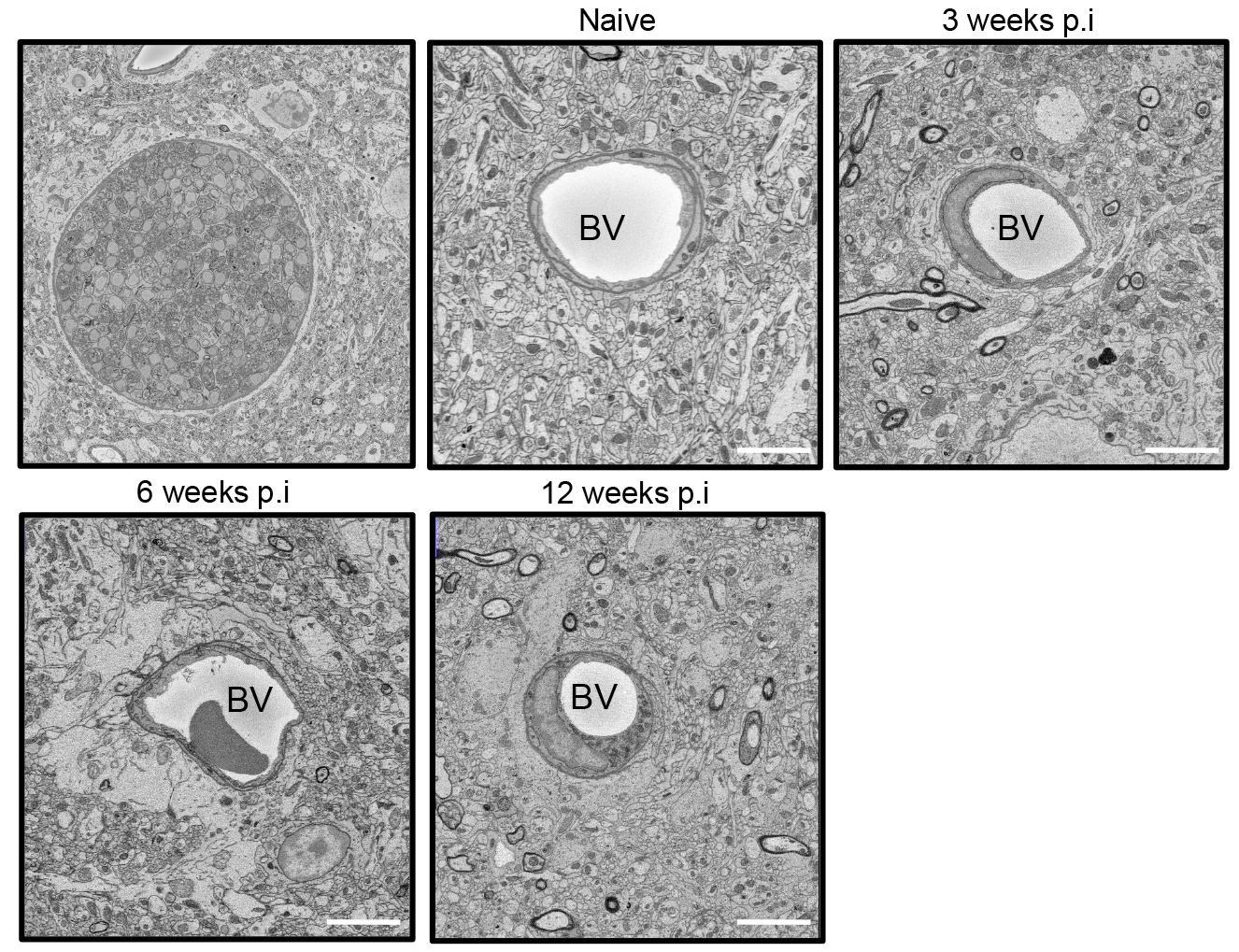

Supplement: S1 Fig — C57Bl/6 mice were infected with Toxoplasma and brains harvested. Scanning serial electron microscopy images analyzed for astrocytic endfeet width (Fig 1). Micrographs pictured here without highlighted borders for clarity. (TIF) [file ppat.1005643.s001.tif]

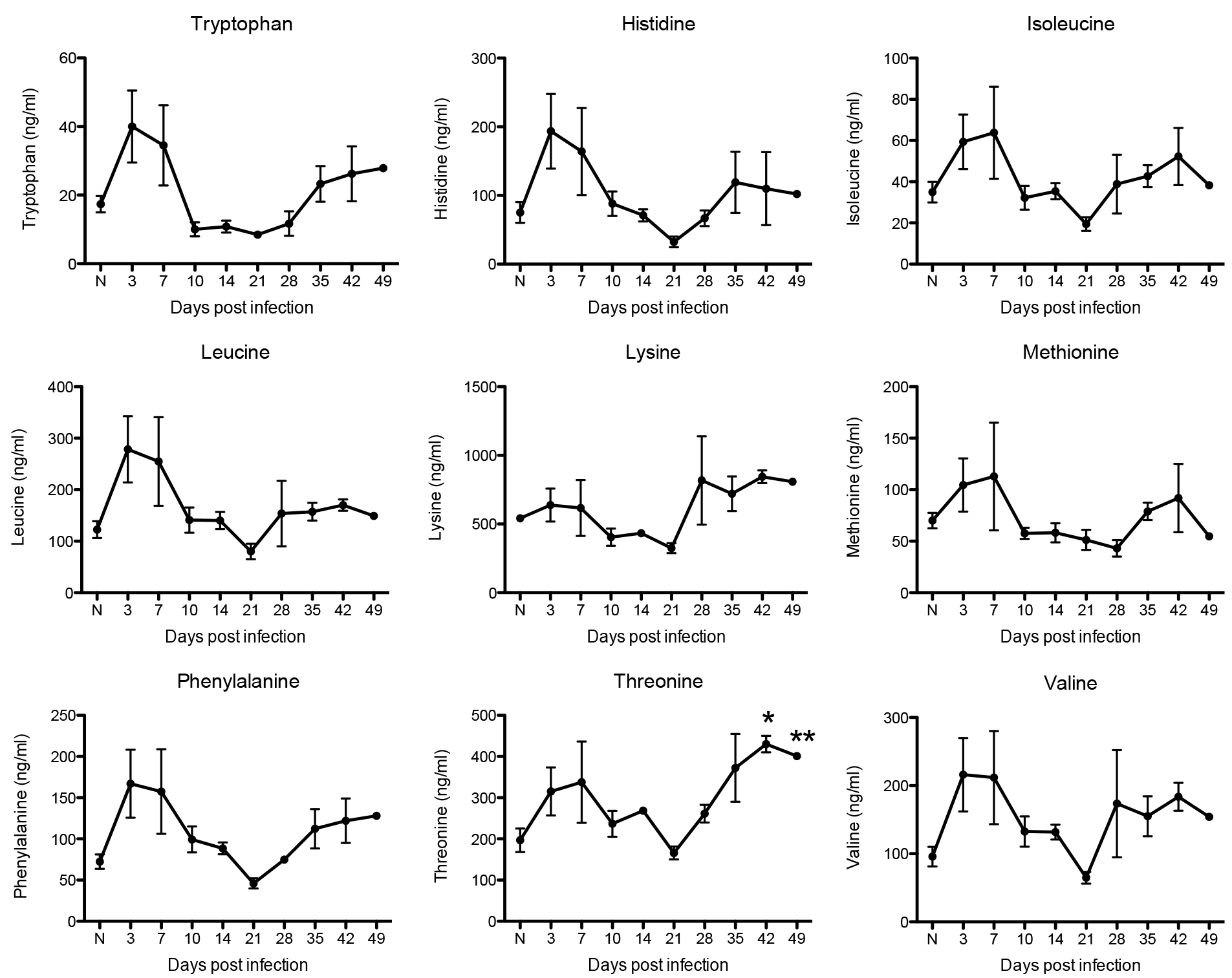

Supplement: S2 Fig — Microdialysis was performed over the course of Toxoplasma infection taking measurements prior to (N) and after infection as indicated (n = 13 biological replicates (3 prior to infection; 2 for each time point thereafter)). LC-MS analysis on microdialysis samples over the course of infection. A one-way ANOVA: Tryptophan (p = 0.0071), Histidine (p = 0.0403), Lysine (p = 0.0243), Phenylalanine (p = 0.0229) and Threonine (p = 0.0052). A Dunnett’s post-test was performed for all timepoints against naïve concentrations and significance shown as asterisks. Amino acids not listed did not change significantly. (TIF) [file ppat.1005643.s002.tif]

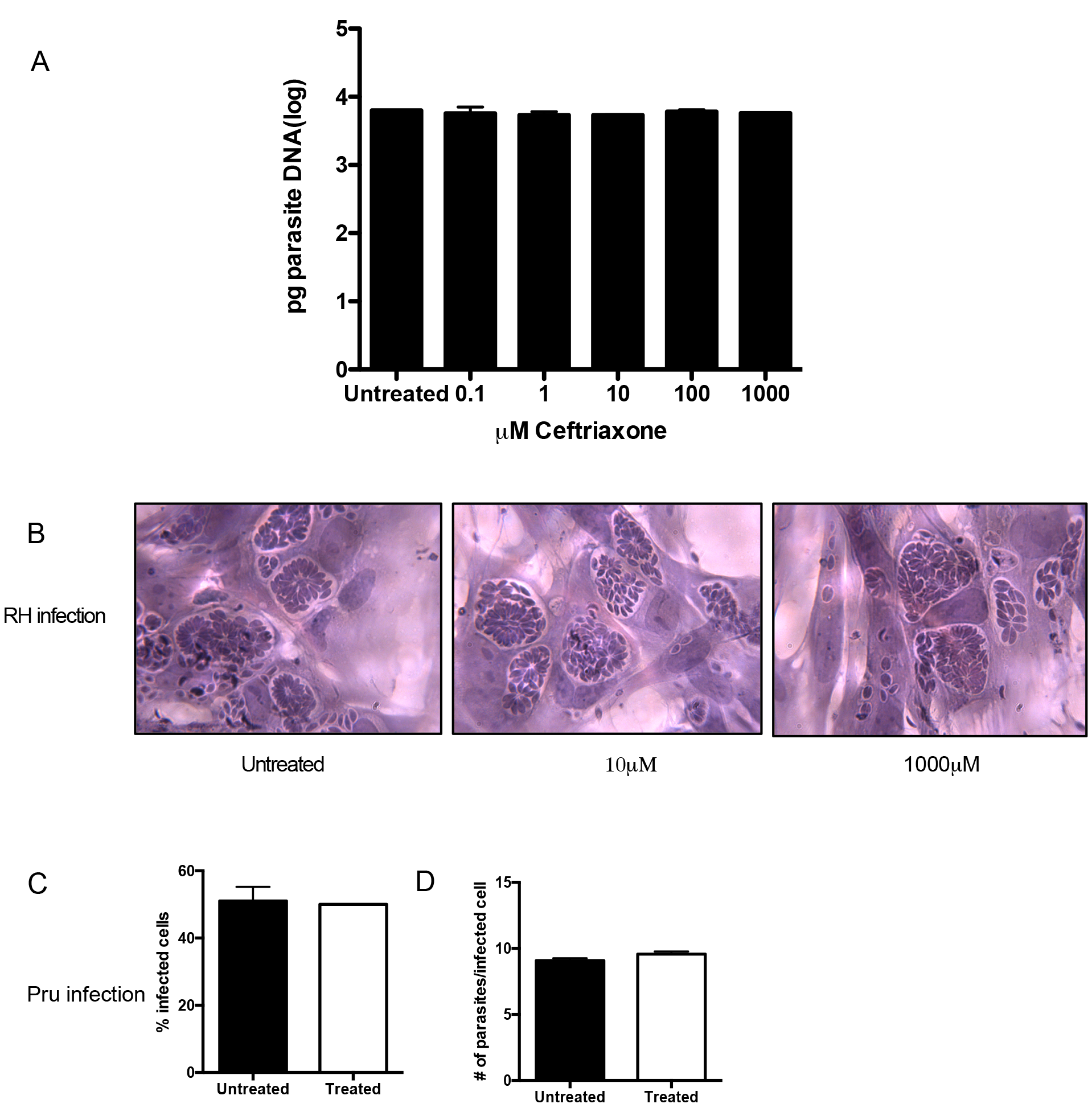

Supplement: S3 Fig — Human foreskin fibroblasts (HFF) were cultured in triplicate and infected with A), C) and D) Prunigund (type II strain parasites) or B) RH (type I parasites) at a MOI of 3:1. Uninvaded parasites were removed after 3hrs, cells were cultured for a further 24hrs. Media was removed and cells were cultured for a further 24hrs with fresh media containing various concentrations of ceftriaxone (0.1–1000μM). A) Infected cells were harvested and RT-PCR was conducted for the Toxoplasma B1 gene. B)-D) cells histologically stained and percentage of cells infected and number of parasites per infected cell quantified from 3 infected wells per group. (TIF) [file ppat.1005643.s003.tif]

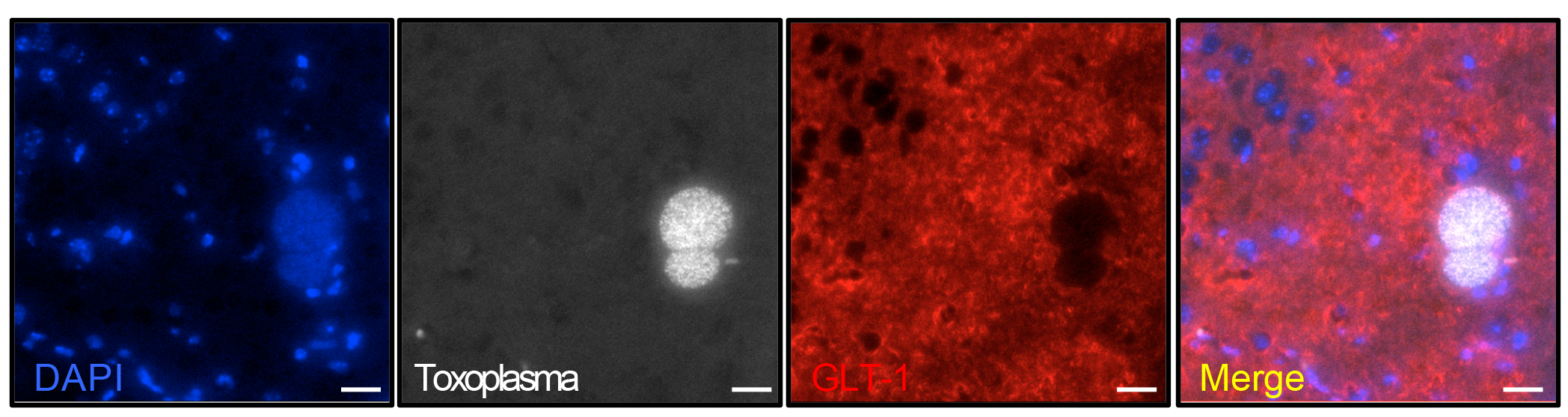

Supplement: S4 Fig — Brain sections from 6 week Me49 infected C57Bl/6 mice were immunohistochemically stained as described in methods. (TIF) [file ppat.1005643.s004.tif]

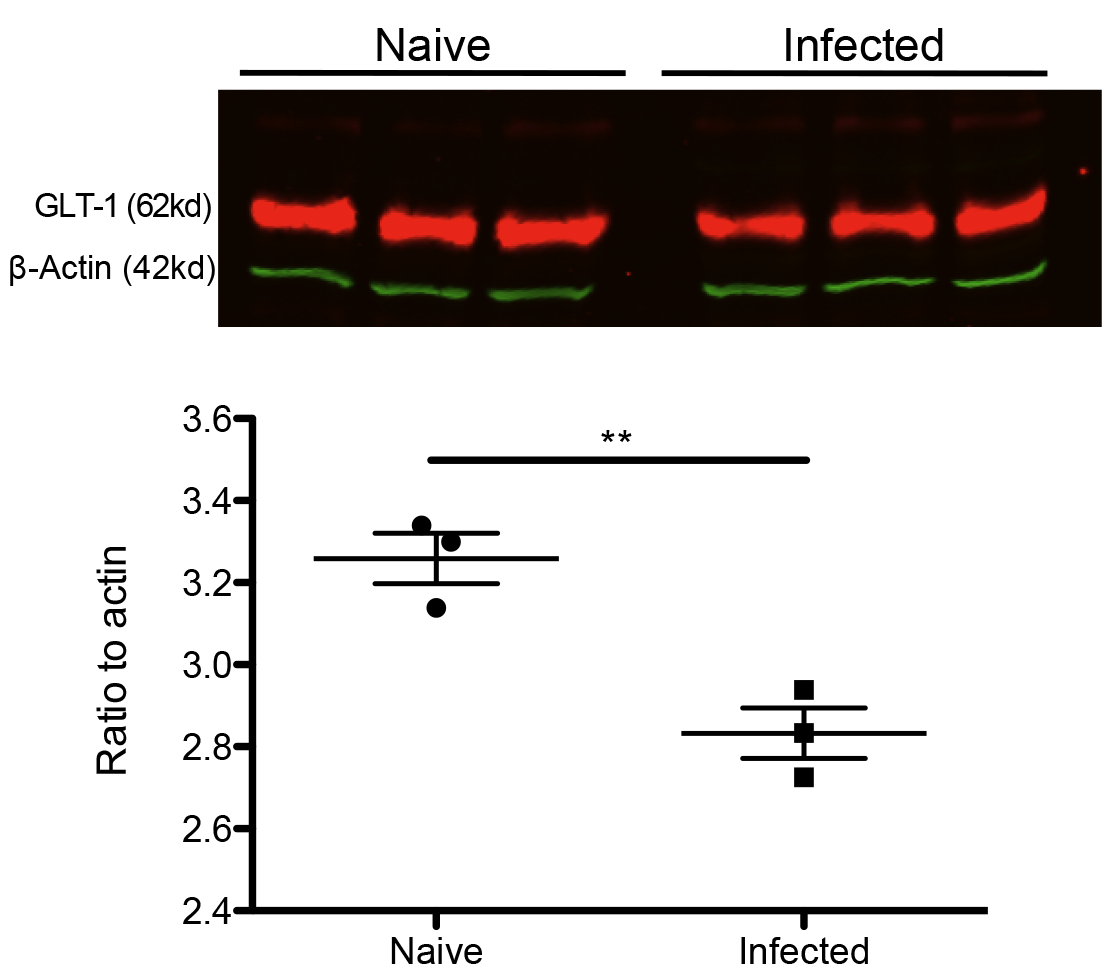

Supplement: S5 Fig — Western blot using protein derived from whole forebrain naïve (n = 3) and infected (n = 3) BALB/c mice were conducted and quantified for GLT-1 (Student’s t-test: p = 0.0081). (TIF) [file ppat.1005643.s005.tif]

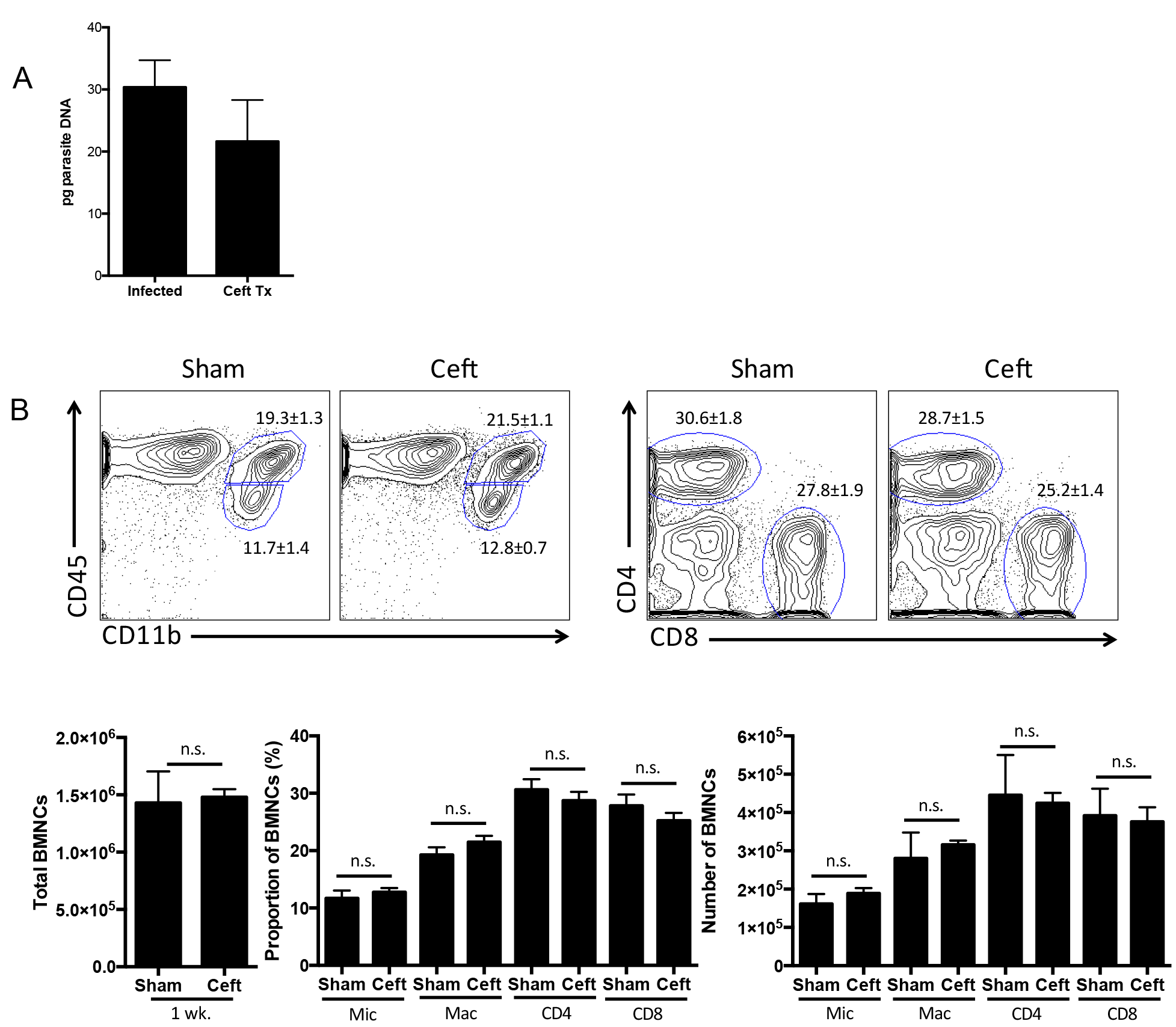

Supplement: S6 Fig — Age matched female C57Bl/6 mice were infected with T. gondii. At 3 weeks post-infection a cohort were treated with ceftriaxone (n = 4) and compared to uninfected naïve control mice (n = 4). A) Parasite burden was quantified using RT-PCR and B) total immune cell infiltration and phenotype were quantified using cell counts and flow cytometry. Using Student’s t-test no significant differences were measured. (TIF) [file ppat.1005643.s006.tif]
